# Recent advances in three-dimensional nanoflower-based membrane engineering for water and wastewater treatment: from fundamental mechanisms to scale-up

**DOI:** 10.1039/d6ra04768h

**Published:** 2026-10-02

**Authors:** Sama Khalili Moradkhanloo, Ratul Kumar Das, Siu-Ning Leung, Satinder Kaur Brar

**Affiliations:** a Department of Civil Engineering, Lassonde School of Engineering, York University Ontario Canada satinder.brar@lassonde.yorku.ca; b Department of Mechanical Engineering, Lassonde School of Engineering, York University Ontario Canada

## Abstract

Three-dimensional (3D) nanoflower (NF)-based membranes have recently emerged as a promising platform for advanced water and wastewater treatment owing to their hierarchical architectures, high surface-to-volume ratio, tunable physicochemical properties, and multifunctional performance. Unlike conventional nanomaterials, NF structures provide abundant exposed reactive sites, interconnected transport pathways, and enhanced interfacial interactions, enabling simultaneous improvements in adsorption, separation, catalytic activity, hydrophilicity, antifouling behavior, and contaminant selectivity. These distinctive features have accelerated their integration into polymeric, ceramic, and thin-film composite membranes for the efficient removal of heavy metals, dyes, pharmaceuticals, nutrients, pathogens, and emerging contaminants. This review critically examines recent advances in engineering 3D NF-based membranes for sustainable water and wastewater treatment, with emphasis on the mechanistic principles governing membrane performance and material functionality. Fundamental transport and removal mechanisms, including adsorption, separation, pore-filling effects, adsorption kinetics, surface charge regulation, wettability modulation, and band-gap engineering for enhanced photocatalytic activity, are comprehensively discussed. Particular attention is given to how NF morphology, composition, and membrane integration strategies influence local membrane properties, transport behavior, permeability-selectivity trade-offs, and long-term operational performance. Recent advances in hybrid NF-incorporated membrane architectures, including metal oxide, metal phosphate as well as nanoflowers integrated with functional materials such as biochar, covalent organic frameworks (COFs), and other functional materials, are systematically evaluated through application-based studies and comparative performance analysis. Critical barriers hindering industrial implementation, including fabrication complexity, structural instability, nanoparticle leaching, membrane fouling, interfacial incompatibility, scalability limitations, and economic constraints, are also discussed under realistic operational conditions. Emerging opportunities in scalable manufacturing, hybrid membrane engineering, computational modeling, and artificial intelligence-assisted materials design are highlighted as pathways toward commercialization. By integrating mechanistic understanding with application-oriented analysis, this review establishes a comprehensive framework for the rational design and large-scale deployment of NF-based membranes, positioning them as promising next-generation technologies for sustainable, energy-efficient, and high-performance water purification systems.

## Abstract 2

Schematic representation of nanoflower-based membranes for water purification, highlighting membrane architecture, nanoflower properties, separation mechanisms, applications, challenges, and fabrication scalability.

## Graphical abstract

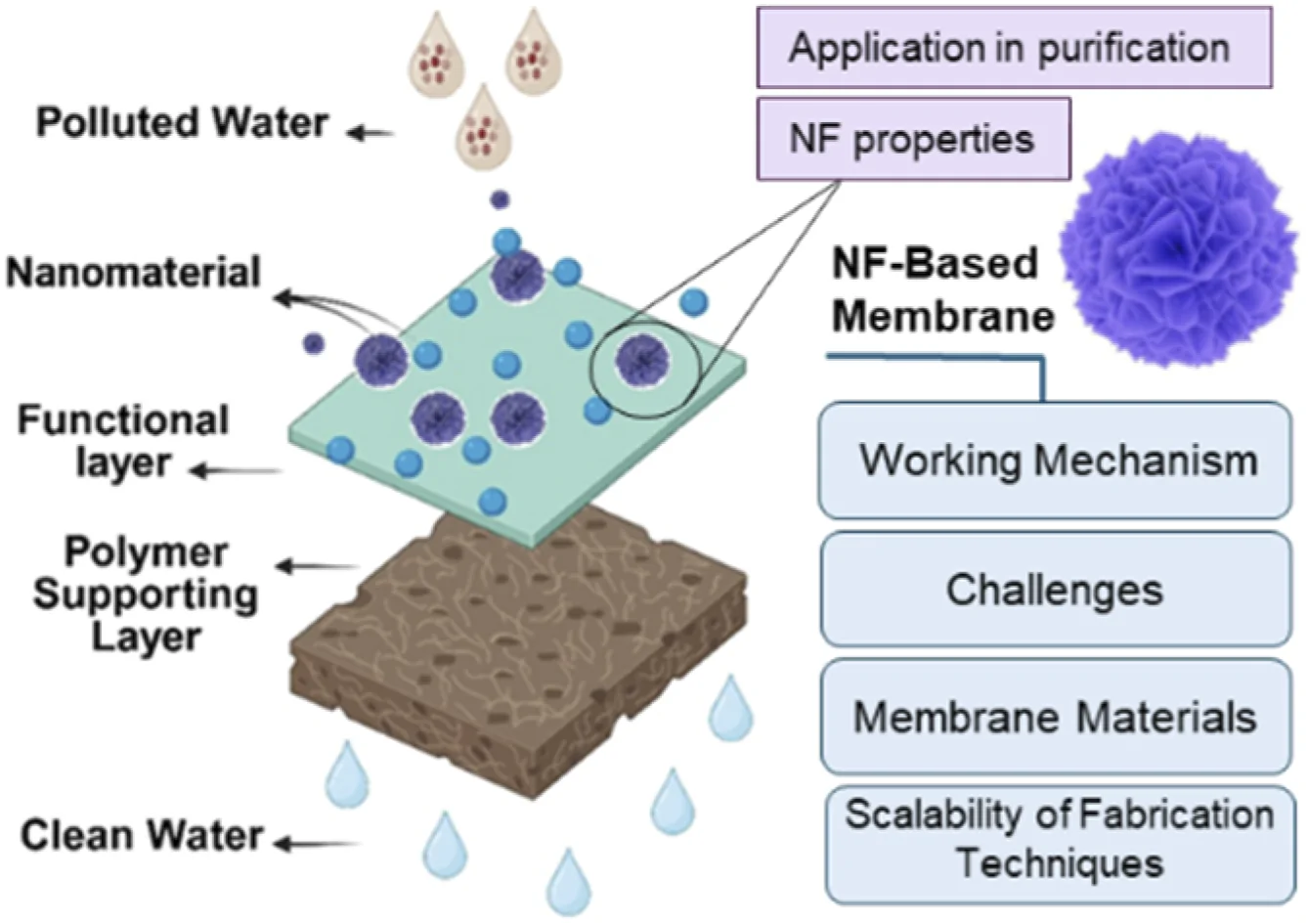

## 1. Introduction

Nanotechnology has emerged as a revolutionary field in modern science, enabling the design, synthesis, and manipulation of nanoscale materials for diverse applications, including healthcare, environmental science, catalysis, and energy storage. Among the various nanostructured materials, nanoflowers (NFs) represent a significant advancement due to their unique three-dimensional (3D) morphology, high surface-to-volume ratio, and enhanced catalytic and adsorption properties. These attributes make NFs particularly attractive for applications in biosensing, drug delivery, wastewater treatment, and energy conversion. The concept of NFs has evolved through extensive research in nanotechnology and materials science. Originally developed as a class of nanostructures that mimic the morphology of natural flowers, NFs have attracted significant attention due to their enhanced physicochemical properties. Early studies focused on the synthesis of inorganic and organic–inorganic hybrid NFs, which subsequently enabled a broad expansion of their applications across biomedical and industrial fields.^1–3^

Subsequent research has focused on improving their synthesis methods, optimizing their structural features, and broadening their practical applications.^3^ NFs exhibit flower-like architectures composed of multiple petal-like nanosheets, providing a high specific surface area and abundant active sites for interaction with target molecules. These structures can be composed of various metal oxides (*e.g.*, zinc oxide, copper oxide, iron oxide), biomolecules (*e.g.*, proteins, enzymes), or hybrid organic–inorganic materials.^4,5^ The composition of NFs determines their functional properties, such as catalytic activity, stability, and biocompatibility, making them suitable for diverse applications in medicine and environmental science^6^ NFs offer several advantages that make them superior to other nanostructured materials. Examples from recent studies clearly demonstrate why NFs outperform many other nanostructured materials. For instance, ZnO NFs show significantly higher adsorption capacities for heavy metals compared to spherical or rod-shaped ZnO nanoparticles due to their multilayered petal architecture that exposes more active sites. Cu_3_(PO_4_)_2_ NFs exhibit superior catalytic activity in phosphatase-mimicking reactions because their hierarchical surfaces facilitate rapid substrate diffusion and strong surface complexation, a performance not achievable with conventional nanoparticle clusters. In photocatalysis, TiO_2_ NFs demonstrate enhanced charge carrier separation and reduced electron–hole recombination relative to TiO_2_ nanotubes or nanospheres, leading to faster degradation rates of organic pollutants. Additionally, enzyme–NF hybrid structures, such as protein–inorganic NFs, provide high enzymatic stability and reusability compared to free enzymes or simple enzyme immobilization on flat substrates. Collectively, these examples illustrate that the hierarchical, high specific surface area, and multiscale porosity of NFs can promote adsorption, catalytic, and photochemical performance compared with many other nanoscale morphologies.^7–13^ The petal-like structure of NFs provides a larger surface area, enhancing their adsorption capacity and catalytic efficiency.^14^ Hybrid NFs incorporating biomolecules demonstrate enhanced stability, making them promising candidates for biomedical applications.^12^ The 3D structure of NFs facilitates better charge carrier mobility, which is crucial for applications such as surface-enhanced Raman spectroscopy (SERS) and photocatalysis.^15^ In this context, NFs are widely used in drug delivery, biosensing, environmental remediation, and energy storage.^16,17^

Application of NFs in water treatment is based on their ability to efficiently remove dyes, heavy metals, and organic pollutants, making them a promising class of materials for sustainable environmental remediation. The development of NFs has been propelled by recent advances in material science, emphasizing structural optimization and functionalization to enhance their performance in water treatment applications.^4,10,18–22^ NFs exhibit interface properties that make them highly effective in water purification. Their high porosity and layered structure facilitate efficient interaction with contaminants, thereby enhancing adsorption and catalytic degradation. Metal oxide-based NFs, such as ZnO, CuO, and Fe_3_O_4_, are widely used due to their photocatalytic properties, enabling the breakdown of complex organic pollutants under visible or UV light.^20,23,24^ Key properties and applications of NFs in water and wastewater purification are summarized in (Fig. 1). Customization of NFs for water treatment involves tailoring their chemical composition, surface functionalization, and hybridization with biomolecules or other nanomaterials to enhance selectivity and efficiency. Table 1 summarizes the classification of three-dimensional nanoflowers according to their composition, morphology, synthesis methods, and membrane integration strategies.

**Fig. 1:**
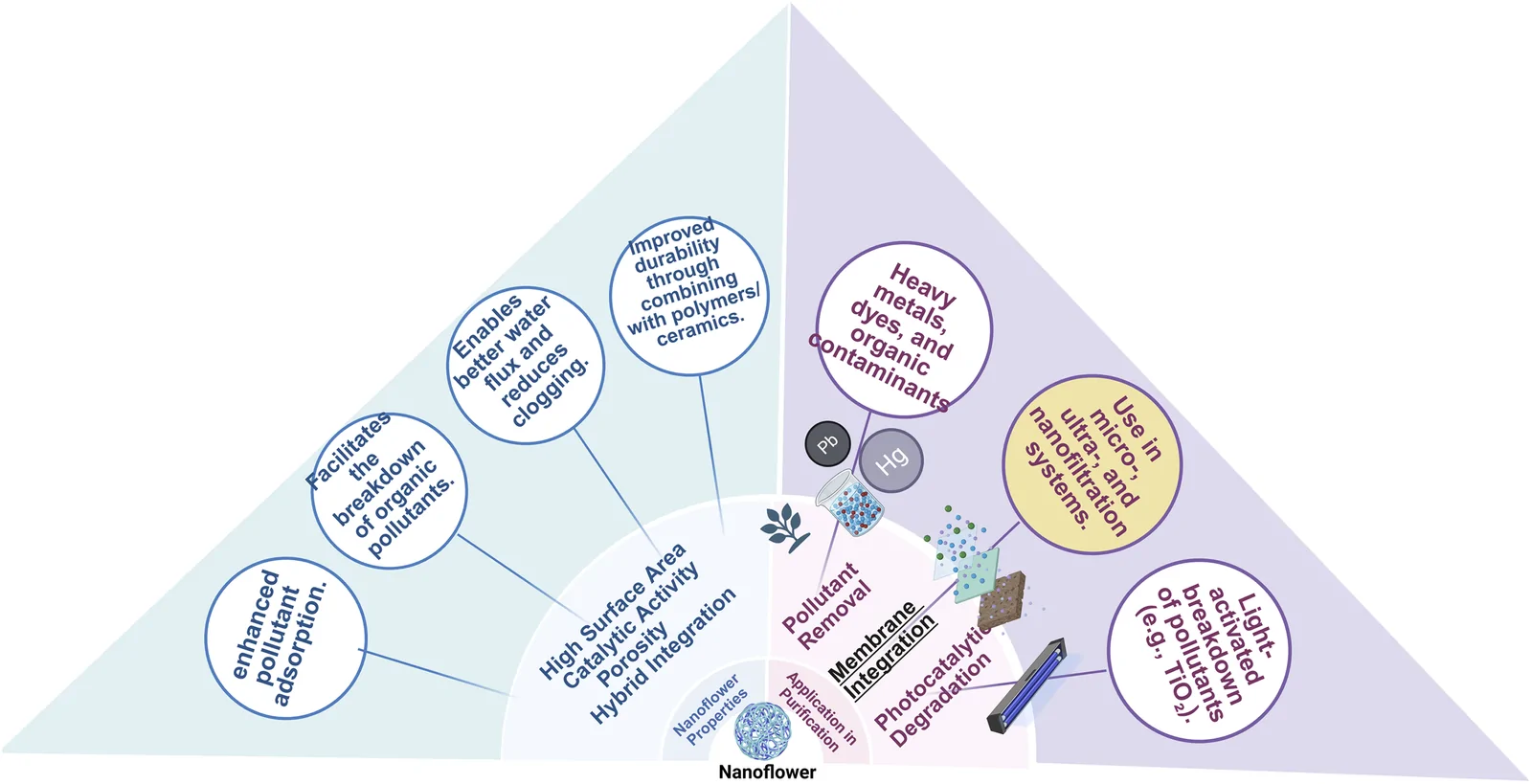
Key properties and applications of NFs in water and wastewater purification.

**Table 1:** Classification of three-dimensional NFs based on composition, morphology, synthesis method, and membrane integration route

Nanoflower class	Representative composition	Typical flower size (µm)	Petal thickness (nm)	Common synthesis method	Membrane integration route	Reference
Metal oxide NFs	ZnO, TiO_2_, CuO, Fe_2_O_3_, MnO_2_, SnO_2_	∼0.2–5	10–100	Hydrothermal, solvothermal, sol–gel, co-precipitation, green synthesis	Blending, surface coating, and interfacial polymerization	7, 10, 21 and 25–29
Metal phosphate NFs	Cu_3_(PO_4_)_2_, Co_3_(PO_4_)_2_, Zn_3_(PO_4_)_2_	∼0.5–10	20–200	Biomimetic mineralization, self-assembly	Surface immobilization, *in situ* growth, mixed-matrix membranes	13 and 30
Layered double hydroxide (LDH) NFs	NiCu-LDH, MgAl-LDH, ZnAl-LDH	∼0.5–3	5–50	Hydrothermal, co-precipitation	Thin-film nanocomposite (TFN), mixed-matrix membranes	14 and 31–33
Metal–organic framework (MOF) NFs	ZIF-8, MIL-series, UiO-based NFs	∼0.3–5	20–100	Solvothermal, self-assembly	Interfacial polymerization, TFN membranes	33
Covalent organic framework (COF) NFs	COF nanoflowers	∼0.2–2	20–80	Solvothermal/interfacial synthesis	Thin-film nanocomposite membranes	3, 20 and 34
MXene-based hierarchical NFs	MXene/NiCu-LDH composites	∼0.5–3	Nanosheet petals	Hydrothermal/self-assembly	Surface deposition, composite membranes	35 and 36
Organic–inorganic hybrid NFs	Protein/Cu_3_(PO_4_)_2_, enzyme/phosphate hybrids	∼1–20	Nanopetal assemblies	Biomimetic self-assembly	Immobilization on porous or nanofibrous membranes	2, 3, 5 and 14

Such modifications facilitate the development of hybrid NFs capable of selectively targeting specific pollutants, thereby improving their overall effectiveness in wastewater treatment.^5,24^

Despite their advantages, the application of NFs in wastewater treatment presents several challenges. One of the main concerns is their reusability and stability, as repeated use may lead to structural degradation and reduced efficiency. Additionally, the cost of large-scale synthesis and potential environmental toxicity associated with the release of nanomaterials into water bodies are critical factors that need to be addressed.^4^

Efforts are being made to develop eco-friendly and cost-effective synthesis methods, such as green synthesis approaches that utilize plant extracts to fabricate NFs with minimal environmental impact.^28^ Furthermore, integrating NFs into membrane systems and composite materials can improve their practical applicability and enhance their durability in water treatment processes.^15^ Further research is required to optimize their synthesis, enhance their stability, and assess their long-term environmental impact. By addressing these challenges, NFs could become a mainstream technology in sustainable water treatment, contributing to global efforts in environmental conservation and resource management.^19,23,37^

Although several recent reviews have discussed nanoflower-based materials or nanocomposite membranes for water treatment, these studies have generally focused on specific material classes, individual membrane processes, or isolated applications. A comprehensive review that systematically examines the relationships among nanoflower morphology, membrane engineering strategies, transport mechanisms, adsorption behavior, photocatalytic activity, antifouling performance, scalability, and environmental sustainability remains lacking. Furthermore, the emerging role of artificial intelligence (AI) and machine learning (ML) in accelerating membrane material discovery and optimization has received limited attention in previous reviews. Therefore, this review provides an integrated and critical perspective on the design, fabrication, characterization, performance, challenges, and future development of three-dimensional nanoflower-based membranes, while identifying current research gaps and future directions toward practical and large-scale water.

The literature included in this review was identified through structured searches of Web of Science, Scopus, ScienceDirect, and Google Scholar. The search focused primarily on publications from 2010 to June 2026 using combinations of keywords including nanoflower, hierarchical nanostructure, nanomaterial membrane, adsorption, photocatalysis, antifouling, combined using Boolean operators (AND/OR). Additional relevant studies were identified through citation tracking of key publications. Studies were selected based on their relevance to nanoflower-based membrane engineering, including synthesis, characterization, membrane integration, separation mechanisms, antifouling behavior, and water and wastewater treatment applications. Publications unrelated to membrane applications, editorials, patents, conference abstracts, and studies lacking sufficient technical information were excluded. The literature identification and selection process is summarized in the PRISMA-style schematic shown in Fig. 2.

**Fig. 2:**
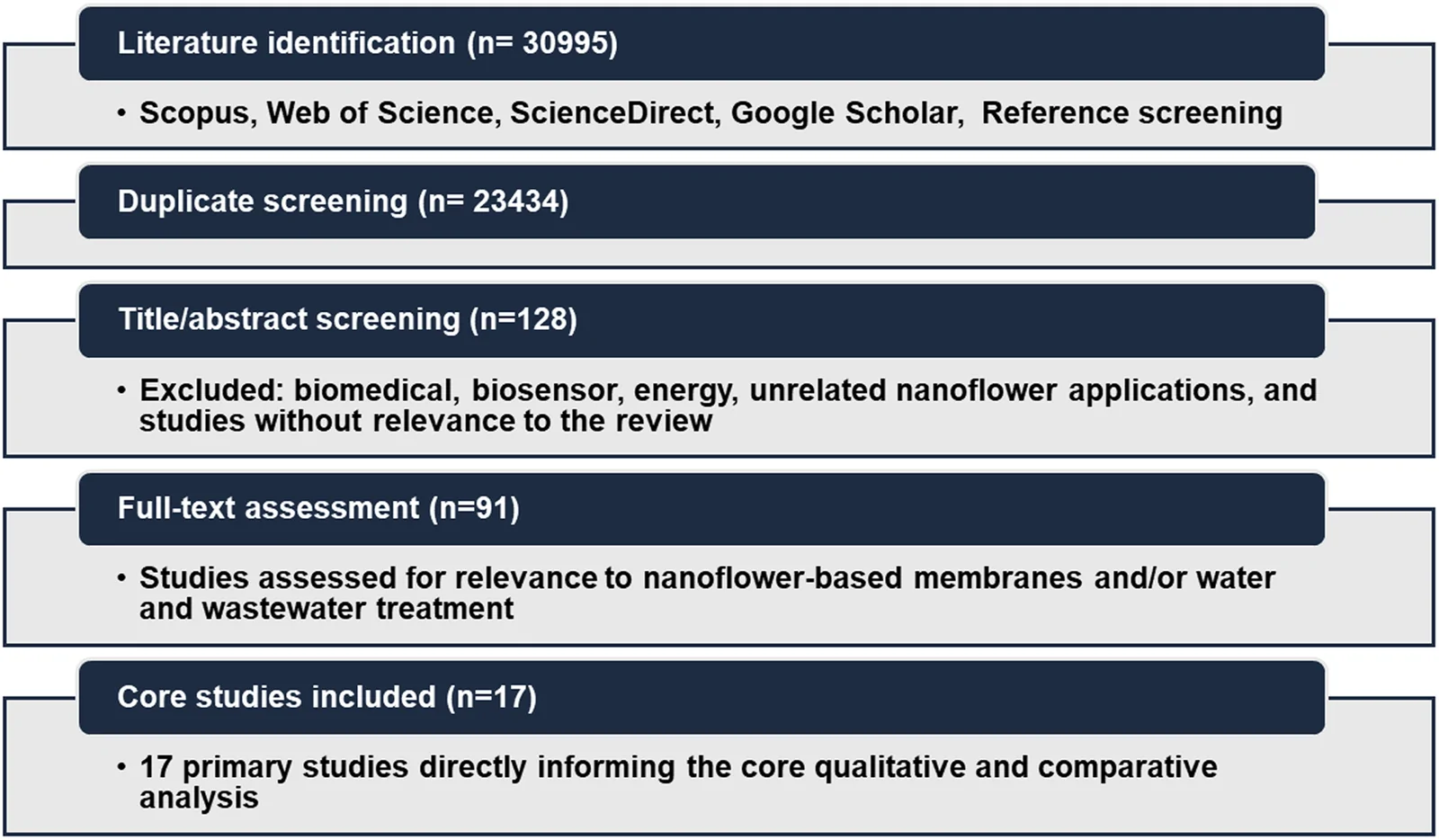
PRISMA-style schematic of the study selection procedure.

Continuous advancements in NF technology have the potential to transform numerous scientific and industrial domains. While NFs show significant promise in water and wastewater purification, further development is required to address existing limitations and ensure their long-term viability in environmental applications. This review provides a critical overview of recent progress and emerging trends in NF-based membranes, covering fundamental concepts and working mechanisms, selection of active materials, structural designs, desirable effects, current challenges, and potential applications in water purification.

## 2. Integration of NFs into membranes

### 2.1. Working mechanism

Nano-enhanced composite membranes (NECMs) operate through two primary mechanisms: adsorption and separation, as illustrated in Fig. 3. The separation mechanism of NECMs is based on the physical structure and permeability of the membrane. Pollutants larger than the membrane's pores are rejected through molecular sieving, while smaller molecules can pass through to deeper layers of the membrane. This approach allows for the effective separation of contaminants during wastewater exudation. The membrane's structural morphology, including its pore size and distribution, plays a significant role in determining separation efficiency. Furthermore, large surface areas and short diffusion pathways contribute to rapid separation and low operational resistance.^23,24,38^

**Fig. 3:**
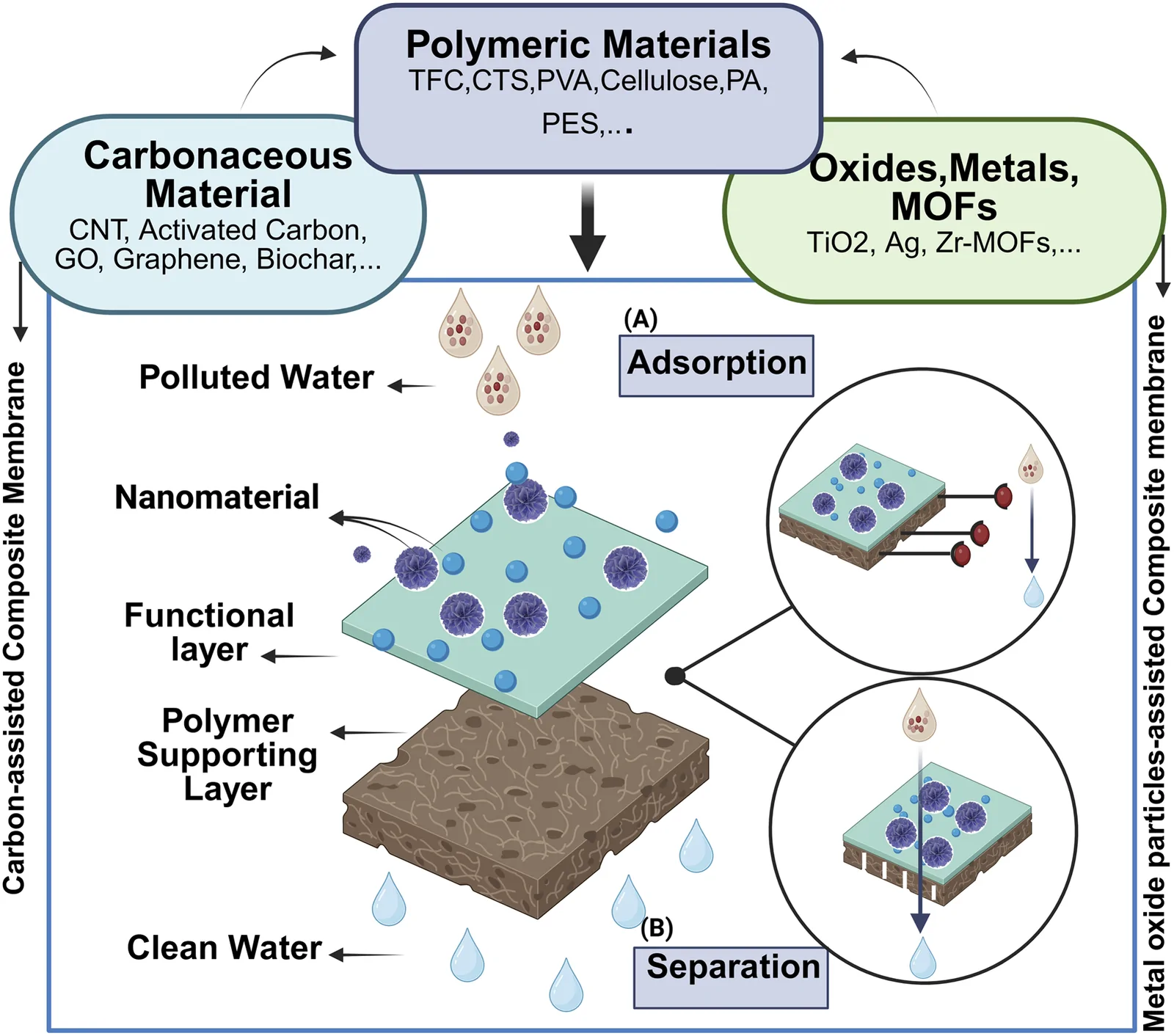
Schematic illustration of NECMs by proposing the incorporation of polymers and nanomaterials, and the working mechanism of NECMs in water/wastewater purification consisting of (A) adsorption (B) and separation.

In adsorption-based removal, NECMs are functionalized with specific chemical groups that interact with and capture target pollutants as water flows through the membrane. This interaction is facilitated by the high surface area and the chemical reactivity of the nanomaterials incorporated into the membranes. The adsorption mechanism allows pollutants to bind to the active sites on the membrane surface or within its pores. The efficiency of this mechanism depends on the proximity between the pollutants and the adsorptive sites, allowing rapid capture at the submicron scale. This enhances the membrane's capacity to remove contaminants through physical and chemical interactions. The pore-filling mechanism involves embedding active nanomaterials directly into the pores of the polymer membrane. This approach enhances both adsorption and separation by providing additional active sites within the membrane structure. Nanomaterials such as Fe_3_O_4_ microspheres and Zr(OH)_*x*_ nanospheres have been successfully incorporated using this method to improve the removal of heavy metals and organic pollutants.

These two mechanisms, adsorption and separation, work together to enhance the filtration performance of NECMs, making them highly effective for removing a wide range of contaminants from water and wastewater.^24^

Importantly, the incorporation of NFs significantly enhances all primary mechanisms of pollutant removal: adsorption and separation. Their unique hierarchical, petal-like morphology and abundant exposed reactive sites, create substantially more adsorption sites than spherical or rod-like nanomaterials, enabling stronger chemisorption, ion exchange, and surface complexation with contaminants. For example, MoS_2_ NFs integrated onto polymer supports exhibit high edge-plane activity, improved charge separation, and increased pollutant affinity, resulting in enhanced adsorption performance. Similarly, hollow FeCoNi-layered double hydroxide (LDH)–Ag NFs provide layered nanosheets and internal cavities that accelerate mass transfer and strengthen electrostatic adsorption of organic pollutants and pesticides. By exposing more functional planes and binding sites, NFs facilitate rapid contaminant capture at both membrane surfaces and internal pore regions, shifting the system toward chemisorption-dominated uptake and improving overall removal efficiency.^38,39^

NFs also influence the separation mechanism by altering membrane surface roughness, hydrophilicity, and polymer cross-linking. When incorporated into thin-film composite (TFC) polyamide (PA) membranes, NFs reshape the ridge-valley topography of the PA selective layer, creating additional nanochannels that facilitate higher water flux while maintaining rejection. This effect is clearly demonstrated in MoS_2_/PDI-modified Janus TFC membranes, where NF infusion decreased contact angle, enhanced hydrophilicity, and increased permeability without sacrificing selectivity. Reported membrane flux and permeance span approximately 350 to over 3200 L m^−2^ h^−1^ among representative NF-based membranes.

Unlike 1D nanorods or 2D nanosheets, the three-dimensional petal-like architecture of NFs introduces steric hindrance that reduces face-to-face stacking and aggregation within the polymer matrix. This 3D morphology promotes more uniform dispersion and minimizes nanoparticle clustering, which in turn reduces macrovoid formation and improves structural homogeneity during membrane fabrication. Likewise, metal oxide NFs such as ZnO and TiO_2_, when embedded within polymer matrices like polysulfone (PSf) or poly vinylidene fluoride (PVDF), reduce foulant adhesion and promote better separation under photocatalytic activation. The hierarchical NF structure contribute to enhanced mechanical stability and more consistent transport pathways during filtration.^40,41^

In addition, NFs substantially improve the pore-filling mechanism when embedded within membrane pores. Their layered or hollow morphologies generate internal transport tunnels and additional active adsorption zones. This was observed in the FeCoNi-LDH NF system, where the hollow NF structure created interconnected porous channels that facilitated diffusion and increased the availability of adsorption sites within the membrane matrix. By occupying polymer voids with reactive NF structures, the membrane gains both structural reinforcement and enhanced chemical activity, which synergistically improve both adsorption and separation efficiency.

Although the incorporation of NF introduces additional adsorption-active sites, adsorption in NF-based membranes does not operate as an isolated process that inevitably leads to fouling. Instead, adsorption works synergistically with size-based filtration and surface-mediated interactions. The hierarchical NF structure enhances contaminant capture while simultaneously improving membrane hydrophilicity and surface charge, which promotes the formation of a hydration layer and reduces nonspecific foulant adhesion. In addition, many NF-modified membranes exhibit antimicrobial or photocatalytic activity, which suppresses biofilm formation and enables partial self-cleaning during operation. As a result, adsorption is often localized and reversible rather than causing irreversible pore blockage, allowing NF-based membranes to achieve enhanced removal efficiency without proportionally increasing fouling propensity.

The type of polymer used in the membrane strongly affects how NFs integrate and perform. In TFC PA membranes, the porous support layer (usually polyethersulfone (PES) or PSf) and the ultra-thin PA selective layer formed *via* interfacial polymerization provide multiple opportunities for NF incorporation. These include before polymerization, within the PA selective layer, or grafted onto the surface. NF deposition onto the support can influence PA growth, leading to denser and more cross-linked selective layers with lower reverse solute flux. Embedding NFs directly into the PA layer creates strong NF–polymer interfacial interactions, enhancing hydrophilicity, catalytic activity, and antifouling properties. This effect has been reported for Au NF that exhibit antimicrobial activity by generating ROS, contributing to reduced biofouling when immobilized on membrane surfaces.

In contrast, polymeric membranes such as PVDF, polyacrylonitrile (PAN), or PSf benefit from NF integration by improving hydrophilicity, enhancing mechanical stability, and creating more efficient water-transport pathways, whereas ceramic membranes gain catalytic synergy and high-temperature/chemical resistance when paired with NFs. Across all membrane types, the integration of NFs consistently enhances rejection performance, increases permeability, and improves antifouling behavior due to their unique structural and chemical features.

It is important to distinguish adsorption from membrane separation and photocatalytic degradation when evaluating membrane performance. Adsorption is governed by the interaction between pollutant molecules and the available active sites on NF surfaces and should therefore be evaluated using adsorption capacity (*q*, mg g^−1^), adsorption kinetics, equilibrium isotherms, and mass-balance analysis rather than rejection efficiency alone. High removal efficiency does not necessarily indicate high adsorption capacity, particularly when pollutant concentrations, adsorbent dosage, or operating conditions differ among studies. Therefore, adsorption performance should be interpreted using standardized adsorption parameters together with mechanistic evidence obtained from kinetic, isotherm, and spectroscopic analyses.^42^

Overall, the incorporation of NFs into NECMs improves adsorption, separation, and pore-filling pathways by integrating high surface area, tunable surface chemistry, and hierarchical porosity with polymeric and TFC membrane architectures. Collectively, these enhancements enable NF-based membranes to outperform conventional nanocomposites and represent a promising direction for next-generation high-efficiency water purification systems.^32,35,40,41,43^

#### 2.1.1 Water flux, porosity, and pore size (separation)

The improvement in flux performance is closely related to the physicochemical properties and morphology of the integrated NFs. Their hierarchical and porous architectures provide additional transport pathways for water molecules, facilitating faster permeation across the membrane surface. Furthermore, NFs with high surface area and functional groups can promote interfacial compatibility with the polymer matrix, minimizing macro void formation and contributing to more uniform contributing to a more uniform pore size distribution of the polymer matrix.

Incorporating NFs or other functional nanostructures into the membrane can also lead to the formation of interconnected networks that enhance water transport channels. This interconnected structure not only improves flux but can also contribute to higher contaminant rejection by balancing permeability and selectivity. Representative NF-based membranes have achieved approximately 95–99.4% contaminant rejection or separation. Overall, NF-modified membranes exhibited improved flux performance due to their high surface porosity, tunable hydrophilicity, and unique three-dimensional morphology, which collectively facilitate efficient water passage through the membrane.

Unlike adsorption, membrane separation is a pressure- or concentration-driven process in which contaminant removal is quantified by permeate flux, solute rejection, and permeate/retentate concentrations. Consequently, membrane performance should be evaluated using water permeance, rejection efficiency, fouling resistance, and long-term operational stability rather than adsorption capacity.^42^

To quantitatively relate the above morphological changes to membrane performance, several structural parameters are commonly evaluated in the literature. Membrane porosity, mean pore radius, and permeability are frequently calculated using established transport models and empirical relationships. These equations provide a quantitative framework for relating NF incorporation to measurable changes in pore structure and water transport behavior. By applying them, researchers are able to assess how variations in NF loading, dispersion, and interfacial compatibility influence the effective pore size distribution and overall membrane permeability. Therefore, eqn (1)–(3) are introduced here to illustrate the quantitative methods typically used to correlate membrane morphology with separation performance.

Water flux refers to the volume of water that passes through a membrane per unit area and per unit time, representing the rate of water permeation. In NF-based membrane systems, water flux is commonly evaluated using a crossflow filtration setup and can be calculated using a standard permeation eqn (1). The incorporation of NF structures into the membrane matrix can significantly enhance hydrophilicity and improve the overall permeability of the system. It may influence the effective network density by disrupting polymer chain packing or modifying local cross-linking efficiency, potentially increasing free volume within the membrane matrix.1
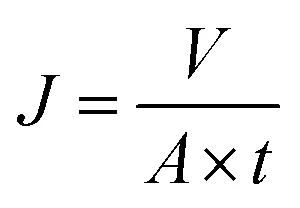
where *J* is the water flux of the membrane, *V* is the liquid volume, *A* is the area of the membrane, and *t* is the specific time.^44^

Profile solution was used as the wetting liquid. The average porosity (*ε*) of the membranes was determined using the gravimetric method, as shown in eqn (2):2
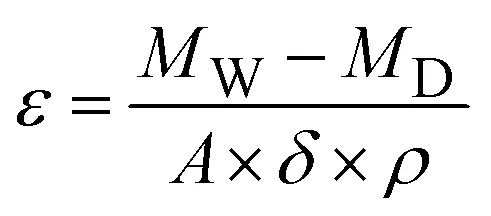
where *M*_W_ and *M*_D_ (g) are the masses of the wet and dry membranes, *A* (cm^2^) is the effective membrane cross-sectional area perpendicular to the direction of flow, *δ* (cm) is the membrane thickness, and *ρ* (1.87 g cm^−3^) is the density of the profile solution.^45^

The mean pore size (*r*_m_) of the membranes was calculated using the Guerout–Elford–Ferry equation based on porosity and pure water flux, as given in eqn (3):3
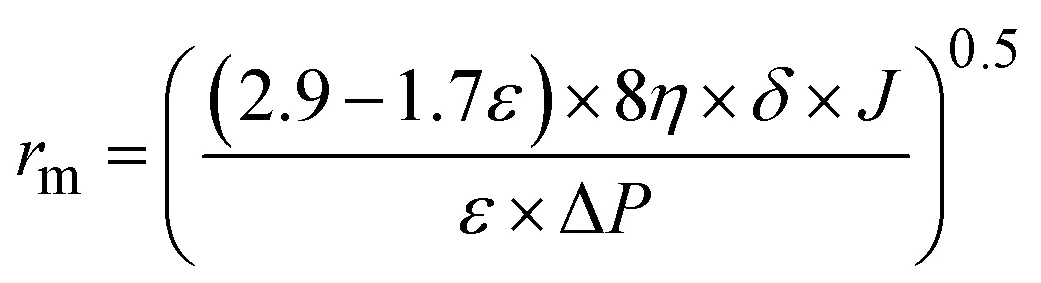
where *η* is the viscosity of water (8.9 × 10^−4^ Pa s), and Δ*P* (MPa) is the operating pressure.^46^

#### 2.1.2 Adsorption kinetics

In most NF-based membranes, the adsorption process is best described by the pseudo-second order (PSO) kinetic model. This is primarily because NFs possess highly reactive surfaces, abundant functional groups, and hierarchical porous structures that promote chemisorption-dominated interactions. The uptake of contaminants on NFs typically involves electron sharing, surface complexation, ion exchange, or coordination with exposed metal sites. Although a good fit to the PSO model may suggest that chemisorption contributes to adsorption, kinetic fitting alone is still insufficient to conclusively establish the adsorption mechanism. Confirmation requires complementary analyses, including comparison with alternative kinetic models, residual analysis, and independent spectroscopic or mechanistic evidence. Numerous studies on Cu_3_(PO_4_)_2_ NFs, ZnO NFs, TiO_2_ NFs, and biochar–NF composite membranes consistently show PSO rate constants with correlation coefficients (*R*^2^) exceeding 0.99, confirming that chemical adsorption is the rate-controlling step rather than simple physisorption. Consequently, the PSO kinetic model provides a more accurate representation of pollutant uptake on NF membranes and is widely used to evaluate their adsorption performance. The model can be expressed as:4
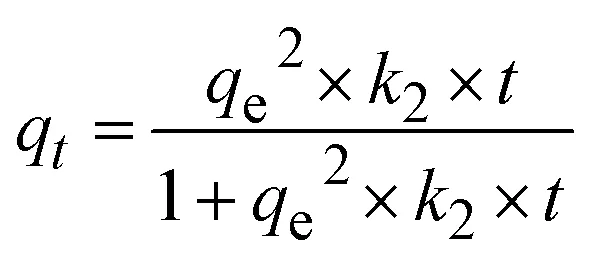
where *q*_*t*_ represents the amount of solute adsorbed on the NF-based membrane at time *t*, *q*_e_ is the equilibrium adsorption capacity, and *k*_2_ is the rate constant of the PSO kinetic model.

Adsorption isotherms are used to explain the interaction between adsorbates and adsorbents and to compare adsorption capacities under equilibrium conditions. The equilibrium adsorption amount can be calculated from the difference between the initial and equilibrium concentrations of the solute as:5
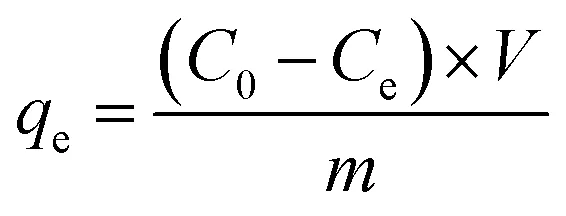
where *C*_0_ (mg L^−1^) is the initial concentration of the adsorbate in solution, *C*_e_ (mg L^−1^) is the equilibrium concentration of the adsorbate in solution, *V* (L) is the volume of the aqueous solution, and *m* (g) is the mass of the adsorbent.

Although the PSO kinetic model and the Langmuir isotherm provide accurate descriptions for many NF-based membranes, the validity and parameters of these models are highly sensitive to the physicochemical characteristics of the NFs. Variations in NF composition, such as differences between metal oxides, metal phosphates, and hybrid biochar–NF structures, directly influence the rate constant *k*_2_ in eqn (4) by altering the density of reactive sites, surface charge distribution, and electron-donating or electron-accepting capabilities. For example, NFs with higher metal oxidation states or stronger Lewis's acidity typically exhibit faster chemisorption, producing larger *k*_2_ values and steeper *q*_*t*_–*t* curves. Similarly, structural modifications including petal thickness, pore connectivity, and crystallinity can change diffusion pathways and activation energies for surface complexation, thereby affecting both *q*_e_ and the overall kinetics.

The Langmuir model frequently provides a satisfactory description of equilibrium adsorption in many NF-based membrane systems. However, a good Langmuir fit should not be interpreted as definitive evidence of homogeneous monolayer adsorption, since multiple adsorption mechanisms may produce similar mathematical behavior. More robust mechanistic interpretation requires comparison with competing isotherm models, statistical evaluation of model fitting, and complementary surface characterization. Studies on Cu_3_(PO_4_)_2_ NF, ZnO NFs, TiO_2_ NFs, and polymer–NF composite membranes consistently report higher correlation coefficients (*R*^2^ > 0.98) for the Langmuir model compared to the Freundlich or Temkin, indicating that adsorption occurs on energetically homogeneous sites. Although Freundlich is occasionally used for heterogeneous systems such as biochar–NF composites, the Langmuir model remains the most accurate for describing equilibrium adsorption on NF surfaces.

The Langmuir isotherm assumes monolayer adsorption on a homogeneous surface and is defined as:6
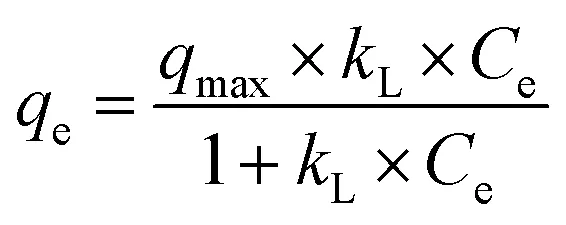
where *k*_L_ and *q*_max_ represent the Langmuir equilibrium constant and the maximum adsorption capacity.

The Freundlich isotherm, which describes multilayer adsorption on heterogeneous surfaces, can be expressed as:

7 *q*_e_ = *k*_F_ × *C*_e_^1/*n*^

where *k*_F_ and 1/*n* are the Freundlich constant and the heterogeneity factor of the adsorption process.^21,47^

The equilibrium parameters in the isotherm equations are equally dependent on NF type. In eqn (6), both *q*_max_ and *k*_L_ reflect the intrinsic uniformity and binding affinity of NF active sites. Metal-phosphate NFs, which possess well-ordered coordination environments, often yield higher *q*_max_ values due to energetically homogeneous surfaces conducive to monolayer adsorption. In contrast, hybrid systems such as biochar–NF composites introduce heterogeneous surface chemistry, causing deviations toward Freundlich behavior and altering the empirical constants *k*_F_ and 1/*n* in eqn (7). Additionally, the presence of functional groups, defect sites, or dopants in engineered NFs can shift *C*_e_–*q*_e_ relationships by modifying electrostatic attraction, π–π interactions, or ion-exchange equilibria.

Other factors, such as pH, ionic strength, competing ions, and the degree of NF agglomeration, also exert substantial influence on the model parameters by changing surface charge states, site accessibility, and diffusional resistance. These multi-level dependencies demonstrate that while PSO and Langmuir models reliably fit NF systems, the underlying constants are not universal and must be interpreted in the context of NF morphology, composition, and interfacial chemistry. A more mechanistic integration of NF structure–property relationships into these models is essential to advance predictive adsorption modeling for NF-based membranes.

Although the PSO and Langmuir models are frequently reported for NF-based membranes, their widespread application should be interpreted cautiously. Most studies rely solely on high correlation coefficients (*R*^2^) to identify adsorption mechanisms without evaluating alternative kinetic models, residual distributions, or statistical uncertainty. Furthermore, adsorption behavior varies considerably with NF composition, membrane architecture, feed chemistry, and operating conditions, making direct comparison between studies difficult. Future investigations should incorporate nonlinear regression, uncertainty analysis, and spectroscopic characterization to establish more reliable structure–property relationships and clarify the dominant adsorption mechanisms.

#### 2.1.3 Hydrophilicity or hydrophobicity (contact angle)

Incorporation of NF structures onto membrane surfaces is widely recognized as an effective strategy for tailoring membrane hydrophilicity. The unique hierarchical micro-and-nanostructures of NFs increase the surface roughness and expose a larger number of active sites, which promotes rapid water spreading and results in smaller water contact angles. When combined with hydrophilic functional groups, either intrinsic to the NFs or introduced through additional surface modification, membrane can achieve significantly enhanced wettability. This improvement arises from the synergistic interaction between the three-dimensional NF morphology, which provides abundant pathways for water affinity, and the hydrophilic chemistry, which facilitates the formation of a stable hydration layer. Enhanced hydrophilicity not only accelerates water permeation but also suppresses foulant adhesion, making NF-modified membranes highly advantageous for water purification and wastewater treatment applications where high flux and antifouling performance are critical.^34^

Although NF incorporation markedly improves membrane hydrophilicity, additional modifications are required to ensure stable and durable wettability under practical operating conditions. Hydration layers generated by NF surfaces may diminish over time due to foulant shielding, polymer chain relaxation, or partial dissolution of metal-based NFs, leading to gradual recovery of hydrophobic characteristics. Therefore, strategies such as covalent tethering of NFs to membrane surfaces, grafting of robust hydrophilic moieties, and construction of multi-layered interfacial architectures are essential for preserving long-term hydrophilicity. Moreover, the hierarchical surface roughness introduced by NFs must be carefully controlled, as excessive surface asperities may promote localized fouling or induce undesirable wetting transitions. Advanced modifications, including zwitterionic functionalization, stable oxide-shell coatings, and hydrophilic polymer brushes, can further enhance interfacial water structuring and suppress foulant adsorption. Overall, effective hydrophilicity optimization in NF-based membranes requires not only introducing highly wettable NFs but also the engineering of chemically stable, strongly anchored, and foulant-resistant interfacial layers capable of maintaining low contact angles during extended membrane operation.

#### 2.1.4 Surface charge property

Surface charge plays a crucial role in governing the interfacial behavior and separation performance of NF-based membranes. The incorporation of NF structures frequently alters membrane's zeta potential due to the presence of abundant functional groups and the increased surface roughness associated with their hierarchical morphology. When NFs are deposited onto a membrane surface and combined with a hydrophilic modification layer, a substantial change in surface charge is typically observed. In many systems, the pristine substrates exhibit a relatively negative zeta potential, whereas the introduction of NFs followed by a secondary hydrophilic coating further increases the negative charge, as illustrated in Fig. 4. This enhancement is attributed to the exposure of polar groups and the improved NF dispersion across the membrane surface, together producing a more uniformly charged interface. An increased negative surface charge strengthens electrostatic repulsion toward negatively charged pollutants, suppresses fouling, and improves separation efficiency. These findings highlight the importance of surface charge engineering in NF-modified membranes and demonstrate how hierarchical nanostructures combined with hydrophilic layers can synergistically optimize zeta potential for advanced water treatment applications.^20,48^

**Fig. 4:**
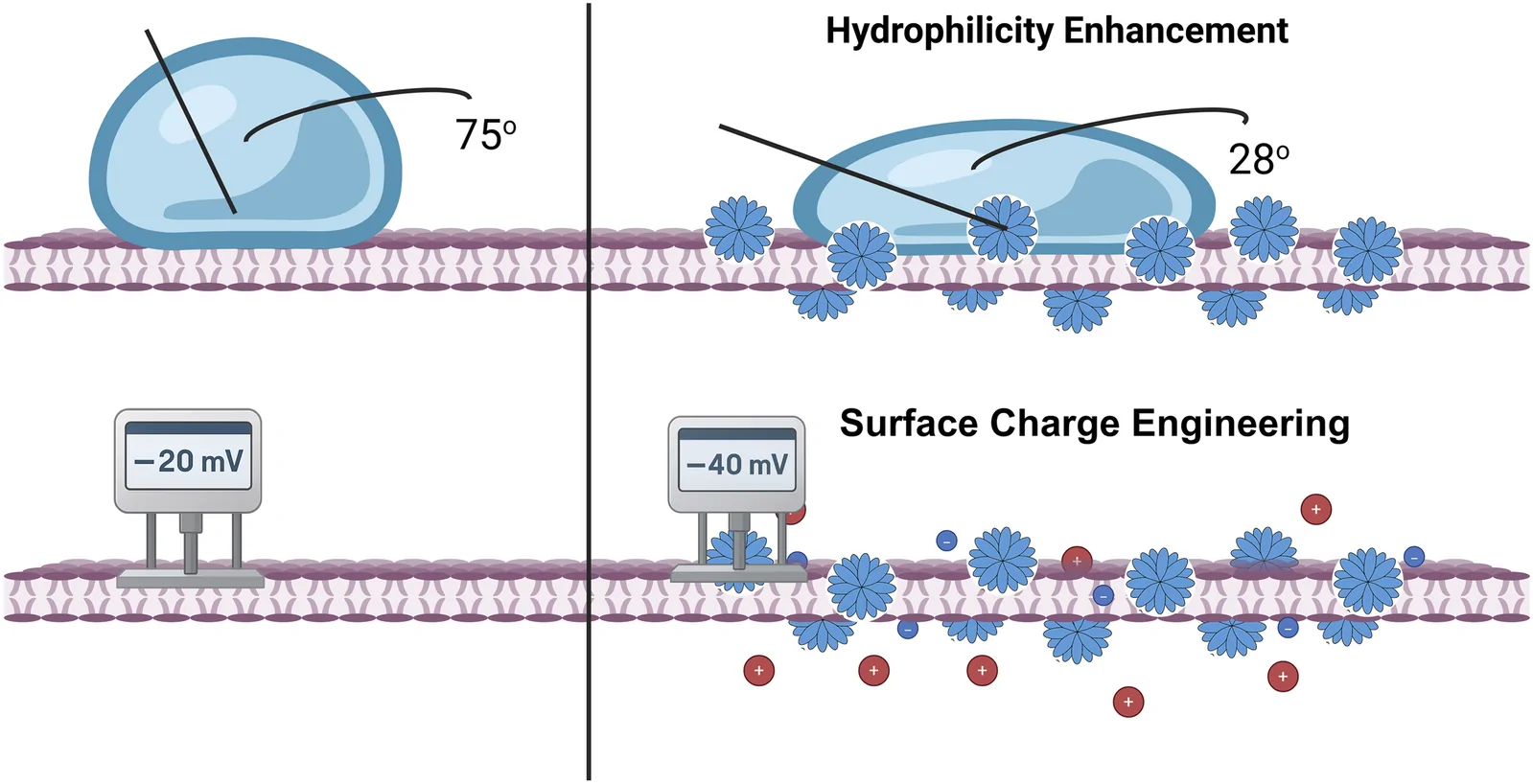
Dual mechanisms of NF-modified membranes: hydrophilicity enhancement and surface charge engineering.

#### 2.1.5 The energy band gaps of NF

The energy band gap (*E*_g_) of NFs is a critical parameter governing their optical absorption, photocatalytic activity, and electron–hole separation efficiency. Owing to their hierarchical architectures composed of ultrathin nanosheets or nanopetals, NFs exhibit electronic structures that differ significantly from bulk materials. This morphology induces quantum confinement, surface defects, and lattice distortion, which collectively contribute to tunable band-gap behavior and directly impact membrane-related applications such as photocatalysis-assisted filtration, self-cleaning, and pollutant degradation.^49–51^

Recent studies demonstrate that band-gap engineering in NFs can be systematically achieved through strategies such as cation doping, facet manipulation, and heterostructure construction. For example, δ-MnO_2_ NFs doped with Mg^2+^, Na^+^, and K^+^ ions exhibit substantial bandgap narrowing (Mg=MnO_2_: 1.08 eV; Na–MnO_2_: 0.90 eV; K–MnO_2_: 0.76 eV), compared with the reported ∼2.3 eV for undoped MnO_2_. The increasing ionic radius of the dopants (Mg^2+^ < Na^+^ < K^+^) promotes electron donation into MnO_2_ layers and stabilizes interlayer spacing, thereby reducing the excitation energy required for electron transition from the valence to conduction band. This tunability enhances visible and near-infrared light absorption, enabling effective activation under low-energy irradiation. Consequently, such band-gap manipulation significantly increases photothermal and photocatalytic efficiencies, as demonstrated in the doped MnO_2_ NF systems.^50^

Band-gap tuning has also been demonstrated in Sn_1−*x*_Ce_*x*_O_2_ NFs, where Ce doping introduces oxygen vacancies and modifies surface electronic states. These structural modifications reduce the band gap and enhance surface-enhanced Raman scattering (SERS) activity by enabling stronger charge-transfer interactions between analytes and the semiconductor surface. In addition, the hierarchical NF morphology exposes a high density of active sites, where Ce-induced defect states facilitate more efficient electron–photon coupling, thereby amplifying optical responses.^49^

Similarly, BiOBr NFs with tunable (001) facets display band gaps ranging from 2.51 to 2.70 eV, depending on the dominant exposed facet. By adjusting synthesis parameters, preferential growth of nanosheets with high-energy surfaces can be achieved, thereby modulating the electronic band structure. BiOBr NFs with a larger proportion of exposed (001) facet exhibit narrower band gaps and enhanced photocatalytic degradation of organic dyes. Their hierarchical, flake-like petals promote rapid charge carrier separation and suppress recombination, which are critical for oxidative degradation reactions.^51^ Moreover, the combined influence of ultrathin nanosheet curvature, vacancy-induced mid-gap states, and inter-petal electronic coupling in NFs significantly narrows the bandgap, as illustrated in Fig. 5, enabling more efficient electron excitation and enhanced photocatalytic performance.

**Fig. 5:**
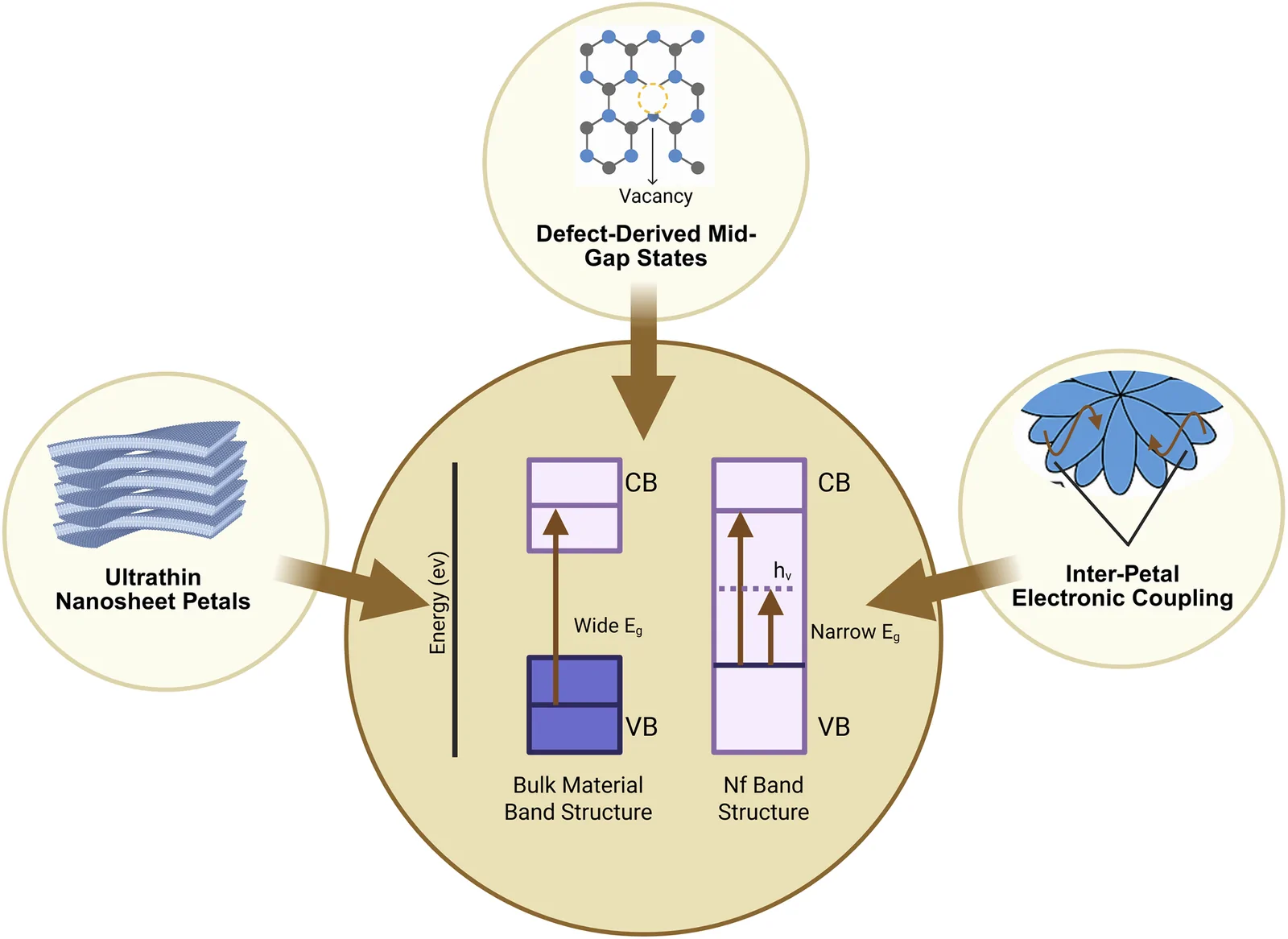
Key mechanisms-nanosheet petals, defects, and electronic coupling-that narrow the band gap in NFs.

Photocatalytic degradation differs fundamentally from both adsorption and membrane rejection because contaminants are chemically transformed rather than merely retained or accumulated. Accordingly, photocatalytic performance should ideally be evaluated using mineralization efficiency (*e.g.*, TOC removal), catalyst stability, reactive oxygen species generation, and identification of transformation products, in addition to apparent degradation efficiency.^42^

Overall, these findings demonstrate that the band gap of NFs is not fixed but highly tunable, enabling the rational design of NF-based membranes with targeted optical and catalytic properties. In water treatment applications, narrower band gaps enable activation under solar or visible light, improve reactive oxygen species (ROS) generation, support self-cleaning membrane functions, and enable integration with advanced oxidation processes (AOPs). Thus, band-gap engineering represents a powerful strategy for optimizing NFs to achieve enhanced pollutant removal, antifouling performance, and energy-efficient water purification. Narrowing the band gap is commonly considered an effective strategy for enhancing photocatalytic performance as band-gap energy alone does not determine catalytic efficiency. Charge-carrier separation, reactive oxygen species generation, catalyst recovery, and long-term photostability often exert equally important influences on membrane performance. Future studies should therefore evaluate photocatalytic activity using comprehensive metrics, including mineralization efficiency, catalyst durability, and quantum efficiency, rather than relying primarily on band-gap values.

While band-gap engineering remains an important strategy for improving visible-light absorption, photocatalytic performance is typically governed by multiple interrelated factors beyond the band-gap value alone. The relative positions of the conduction and valence bands determine the redox potential available for the generation of reactive oxygen species (ROS), including hydroxyl radicals (˙OH), superoxide radicals (˙O_2_^−^), and singlet oxygen, which are responsible for pollutant oxidation and degradation. In addition, efficient separation of photogenerated electron–hole pairs and suppression of charge-carrier recombination are essential for maximizing photocatalytic activity. Operational parameters, such as light intensity, irradiation wavelength, catalyst loading, and solution chemistry, also significantly influence degradation efficiency. Moreover, photocatalytic performance should ideally be evaluated not only by pollutant removal efficiency but also by mineralization indicators such as total organic carbon (TOC) removal and the identification of intermediate transformation products to ensure complete degradation and minimize the formation of potentially harmful by-products. Catalyst stability and recovery are equally important considerations for practical implementation, particularly for reusable membrane-based photocatalysts. Recent studies, such as that reported by Hassan *et al.* (2025), have demonstrated that integrating adsorption, photocatalysis, and magnetic recovery within multifunctional nanocomposites can simultaneously enhance contaminant removal, facilitate catalyst recycling, and improve long-term operational sustainability, highlighting a promising direction for next-generation nanoflower-based membrane systems.^52^

### 2.2. Technical challenges in fabricating commercial membranes for specific uses: conventional modalities and NF-based membranes

Membrane technology has become a cornerstone of modern separation processes, including desalination, wastewater treatment, and industrial separations. Despite its widespread adoption, the fabrication of commercial membranes for specific uses remains challenging. Key challenges arise from material selection, membrane fouling, structural stability, energy efficiency, and scalability. Addressing these challenges is crucial for developing cost-effective, durable, and high-performance membranes capable of meeting industry demands.

One of the primary challenges in membrane fabrication lies in the selection of materials that deliver optimal performance for specific applications. Polymeric membranes, such as PA, PSf, and PES, are widely used due to their cost-effectiveness and ease of fabrication. However, these materials often exhibit inherent trade-offs between permeability and selectivity, which can limit their overall separation efficiency.^53–55^

To enhance membrane performance, nanomaterials (NMs) have been integrated into polymeric membranes. Carbon nanotubes (CNTs), titania nanotubes (TNTs), and metal oxides like ZnO and TiO_2_ have shown potential in improving membrane characteristics. Despite these benefits, nanomaterial incorporation introduces challenges related to uniform dispersion, potential leaching, and long-term stability. Ensuring robust compatibility between nanomaterials and polymeric matrices therefore remains a major obstacle to the large-scale commercialization of polymer nanocomposite membranes.^3,20,56^

Fouling is one of the most critical challenges affecting membrane performance and operational longevity. It occurs due to the accumulation of organic matter, inorganic scaling, biofouling, and particulate deposition on the membrane surface. Because these fouling mechanisms differ in origin and behavior, they require distinct and targeted mitigation strategies:

(1) Organic fouling: caused by natural organic matter, proteins, and oils. Hydrophilic membrane surfaces and antifouling coatings can reduce organic fouling.^57–59^

(2) Inorganic scaling: results from the precipitation of salts like calcium carbonate and magnesium hydroxide. Anti-scaling agents and pH control can help minimize scaling.^57,60^

(3) Biofouling: arises from the growth of microorganisms and biofilm formation. Silver nanoparticles and antibacterial coatings have shown effectiveness in suppressing biofouling.^12,61^

(4) Particulate fouling: occurs due to the deposition of suspended solids, which can clog membrane pores. Proper pre-treatment, including filtration and coagulation, is essential to reduce particulate fouling.^55,58,62^

Despite advancements in antifouling strategies, completely preventing fouling remains challenging. Therefore, membranes require frequent cleaning and periodic replacement, leading to increased operational costs. Commercial membranes must also withstand high operating pressures and harsh chemical environments. Mechanical deformation and chemical degradation are common issues that limit membrane lifespan. For instance, reverse osmosis (RO) membranes operate under high pressures, which can induce material fatigue and structural failure over time.^63^

Enhancing membrane mechanical strength involves optimization of support layers and the selection of robust materials. Thin-film composite (TFC) membranes with improved interfacial adhesion and reinforced support structures offer enhanced mechanical durability. However, balancing mechanical strength and permeability is challenging, as increased membrane density often leads to have reduced water flux.

Energy consumption is another critical concern in pressure-driven membrane processes, such as RO and nanofiltration. The energy required to overcome osmotic pressure and hydraulic resistance contributes significantly to overall operational costs. While highly permeable membranes with lower resistance can reduce energy consumption, increasing permeability often compromises selectivity.^64,65^

Recent research has focused on designing membranes with high permeability while maintaining adequate salt rejection. Techniques such as modified interfacial polymerization and the incorporation of nanomaterials (*e.g.*, NFs, nanoparticles, and other nanostructured fillers) into membrane matrices have shown considerable promise.

In addition, nanoscale engineering of the selective layer-through controlled pore formation and structural tuning-has been explored to enhance transport efficiency without compromising selectivity. However, translating these innovations to commercial-scale membrane fabrication has advanced significantly, scaling production to industrial levels presents substantial technical and economic challenges.^67^ Key challenges in large-scale membrane production include ensuring batch-to-batch consistency, ensure uniform membrane thickness and defect-free structures, developing scalable coating and deposition techniques, reducing production costs while maintaining quality, and addressing environmental concerns related to membrane disposal and recycling.^7,24,25,66^

Membrane technology has advanced substantially in water treatment and separation processes, utilizing microfiltration (MF), ultrafiltration (UF), and nanofiltration. Despite these advances, fabricating membranes that balance permeability, selectivity, and antifouling performance remains a significant challenge. Conventional polymeric membranes often suffer from fouling, degradation under harsh operating conditions, and limited selectivity.^56^ Integrating NF structures into membranes offers a promising strategy to address these limitations by improving surface properties and enhancing adsorption capacity.^67^

The fabrication of commercial membranes for filtration applications involves several technical challenges, including achieving optimal porosity, stability, and resistance to fouling. Conventional membranes frequently encounter pore clogging, limited mechanical strength, and chemical degradation under harsh operating conditions. Recent advances in NF-based membranes offer promising solutions to address these limitations by leveraging unique hierarchical architectures that enhance filtration efficiency and durability. Incorporation of NFs into various membrane technologies, including PA and ceramic composites, has been widely explored to enhance their antifouling properties and permeability.^4,68^

NFs exhibit a large surface area and superior structural stability, making them promising candidates to improve the selectivity and retention performance of micro-, ultra-, and nanofiltration membranes. Their roles in hybrid membrane systems have been demonstrated in several studies, highlighting their catalytic, adsorptive, and antimicrobial functions in water purification.^37^ Furthermore, the hierarchical morphology of NFs enables enhanced molecular sieving, allowing more effective separation of contaminants from water.^59^

Membrane fouling remains a significant challenge in filtration processes, leading to reduced efficiency and higher maintenance costs. Incorporation of NFs has been shown to mitigate fouling by introducing self-cleaning and antimicrobial properties, thereby extending membrane lifespan and sustaining performance. Moreover, NF-based membranes have demonstrated enhanced water flux and selectivity, which are essential for improving filtration effectiveness across diverse applications.^7^ A comparison of key performance parameters between conventional membranes and NF-based membranes is presented in Table 2.

**Table 2:** Comparison of key parameters between conventional membranes and NF-based membranes

Parameter	NF-based membranes	Conventional membranes
Adsorption efficiency	High (due to larger surface area and porosity)	Moderate (limited by surface structure)
Fouling resistance	Improved (with antifouling agents like Ag, TiO_2_)	Prone to fouling (requires frequent cleaning)
Mechanical strength	Moderate (enhanced with hybrid supports)	High (established materials are more robust)
Fabrication complexity	High (requires precise synthesis and hybridization)	Low (simpler and well-established processes)
Scalability	Challenging (requires advanced synthesis methods)	Easier (standardized mass production)
Cost of production	High (complex synthesis and hybrid integration)	Lower (simpler and cost-effective)
Environmental impact	Potential nanoparticle leaching (needs regulation)	Minimal (fewer safety concerns)
Application versatility	Wide (customizable for specific pollutants)	Limited (fixed design and function)
Stability in harsh conditions	Moderate (requires reinforcement)	High (better tolerance to pressure and pH)

Incorporation of NFs into membrane structures has led to significant improvements in permeability, selectivity, and antifouling performance. However, several unresolved scientific and engineering challenges continue to limit their commercial adoption. A major limitation arises from the discrepancy between laboratory-scale evaluations and the complex physicochemical conditions encountered in real feedwaters. Laboratory studies often rely on single solutes or simplified matrices, whereas full-scale systems are exposed to fluctuating ionic strength, competing anions, natural organic matter, colloids, surfactants, and diverse microbial communities. These constituents can alter electrostatic interactions, destabilize NF–polymer interfaces, and accelerate degradation mechanisms that are typically not captured in short-term controlled studies.

The hierarchical architecture of NFs provides high surface reactivity but also introduces structural vulnerability during long-term filtration. Crossflow shear, pressure fluctuations, and repeated cleaning cycles may deform the petal-like motifs, promote delamination, or induce dissolution. However, mechanistic investigations of these degradation pathways remain limited, and the potential release of NF fragments into permeate streams raises concerns regarding both performance reliability and environmental safety. The absence of standardized protocols to quantify NF leaching under realistic operating conditions therefore represents a critical barrier to regulatory approval and large-scale deployment.

Scalability constitutes another major challenge. Most NF synthesis routes, including hydrothermal mineralization, biomolecule-assisted nucleation, template-guided growth, and multi-step hybridization, are batch-dependent and poorly suited to continuous industrial membrane fabrication. Moreover, NF morphology is highly sensitive to precursor concentration, nucleation rate, solution chemistry, and growth kinetics, all of which are difficult to maintain uniformly across large membrane areas. These sensitivities can lead to variability in pore distribution, surface functionality, and defect density, ultimately undermining the consistency required for commercial membrane modules.

From a materials engineering perspective, interfacial compatibility between NFs and polymer matrices remains insufficiently understood. Adhesion is driven by hydrogen bonding, van der Waals interactions, or covalent bridges; however, stress transfer mechanisms within hybrid layers have not been fully characterized. In the absence of robust interfacial anchoring, NF-modified surfaces may undergo functional deterioration during prolonged operation or aggressive cleaning cycles involving alkaline or acidic environments. Furthermore, the improvement in hydrophilicity and surface charge imparted by NFs may diminish over time due to foulant shielding, surface rearrangement, or pore compaction, potentially compromising long-term membrane performance. These evolving interfacial dynamics highlight the need for *in situ* monitoring tools capable of capturing real-time NF performance during filtration.

Economic and environmental considerations further complicate commercial translation. NF synthesis often relies on high-purity reagents, precise reaction control, and multistep processing, which increase cost and energy demand. In addition, the environmental fate of released NF fragments remains poorly understood. Limited data exists regarding their dissolution behavior, bioaccumulation in aquatic systems, ecotoxicity, and end-of-life disposal, creating significant uncertainty surrounding the overall sustainability of NF-based membrane technologies.

In summary, while NF-enhanced membranes exhibit strong potential at the laboratory scale, their industrial viability requires coordinated advances across multiple fronts. These include developing a mechanistic understanding of NF degradation and leaching, improving interfacial stability under realistic hydraulic and chemical stresses, establishing scalable synthesis routes compatible with industrial membrane manufacturing, and conducting comprehensive environmental and economic assessments. Addressing these fundamental challenges is essential to enable the successful transition of NF-based membranes from laboratory research to reliable full-scale water treatment technologies.

Nanoflower-based materials have demonstrated promising performance in water treatment, with reported separation and degradation efficiencies ranging from approximately 88% to 99.4% in representative studies. Meanwhile, their potential environmental release and long-term stability should also be considered. Dissolved metal release should be distinguished from particulate detachment, as these represent different release pathways and require different analytical approaches. Dissolved metal ions released from copper-containing nanoflowers can be quantified using inductively coupled plasma techniques, such as ICP-OES or ICP-MS, while particulate release can be evaluated using complementary nanoparticle characterization approaches, including particle-size, morphology, and elemental analyses. A mass-balance assessment can further determine the distribution of metals between the treated water, retained material, and released particulate fraction. These assessments are important for determining the environmental stability and practical applicability of nanoflower-based materials.^69^

Current research has predominantly focused on improving membrane performance under controlled laboratory conditions. However, relatively few studies systematically investigate long-term operation under realistic wastewater matrices containing natural organic matter, competing ions, microorganisms, and fluctuating hydraulic conditions. Bridging this gap represents one of the most important challenges for translating NF-based membranes from laboratory research to practical water treatment applications.

### 2.3. Selection of membrane materials (organic or ceramic) for NF integration

The selection of membrane materials plays a crucial role in determining the efficiency, durability, and applicability of NF-based membranes. Both organic and ceramic materials offer distinct advantages, and their selection depends on the targeted operating conditions and separation requirements. Organic polymer membranes, including PA and PAN, are widely used due to their flexibility, ease of fabrication, and cost-effectiveness. However, these materials often suffer from lower mechanical strength and susceptibility to chemical degradation under harsh operating environments.^70,71^

In general, membranes used for water and wastewater purification are divided into three typical types, namely, inorganic membranes, polymeric membranes, and composite membranes (Fig. 6). Inorganic membranes, particularly ceramic membranes, provide superior thermal, chemical, and mechanical stability, making them ideal for aggressive filtration environments. The integration of NFs into ceramic matrices has been shown to improve permeability, fouling resistance, and contaminant removal efficiency. Recent research has highlighted the use of NF-enhanced ceramic membranes in wastewater treatment and pharmaceutical filtration, where they outperform conventional membranes. Ceramic substrates present challenges related to uniform NF anchoring due to their rigid microstructure. NF adhesion on ceramic surfaces depends strongly on interfacial chemistry, including metal–oxygen coordination, surface hydroxylation, and charge matching. Insufficient interfacial binding energy can result in NF detachment or fragmentation during backwashing, high-shear crossflow, or aggressive cleaning cycles, raising concerns related to performance degradation and nanoparticle release. However, systematic studies quantifying adhesion strength, interfacial degradation mechanisms, or ceramic-NF compatibility remain scarce.^24,72^

**Fig. 6:**
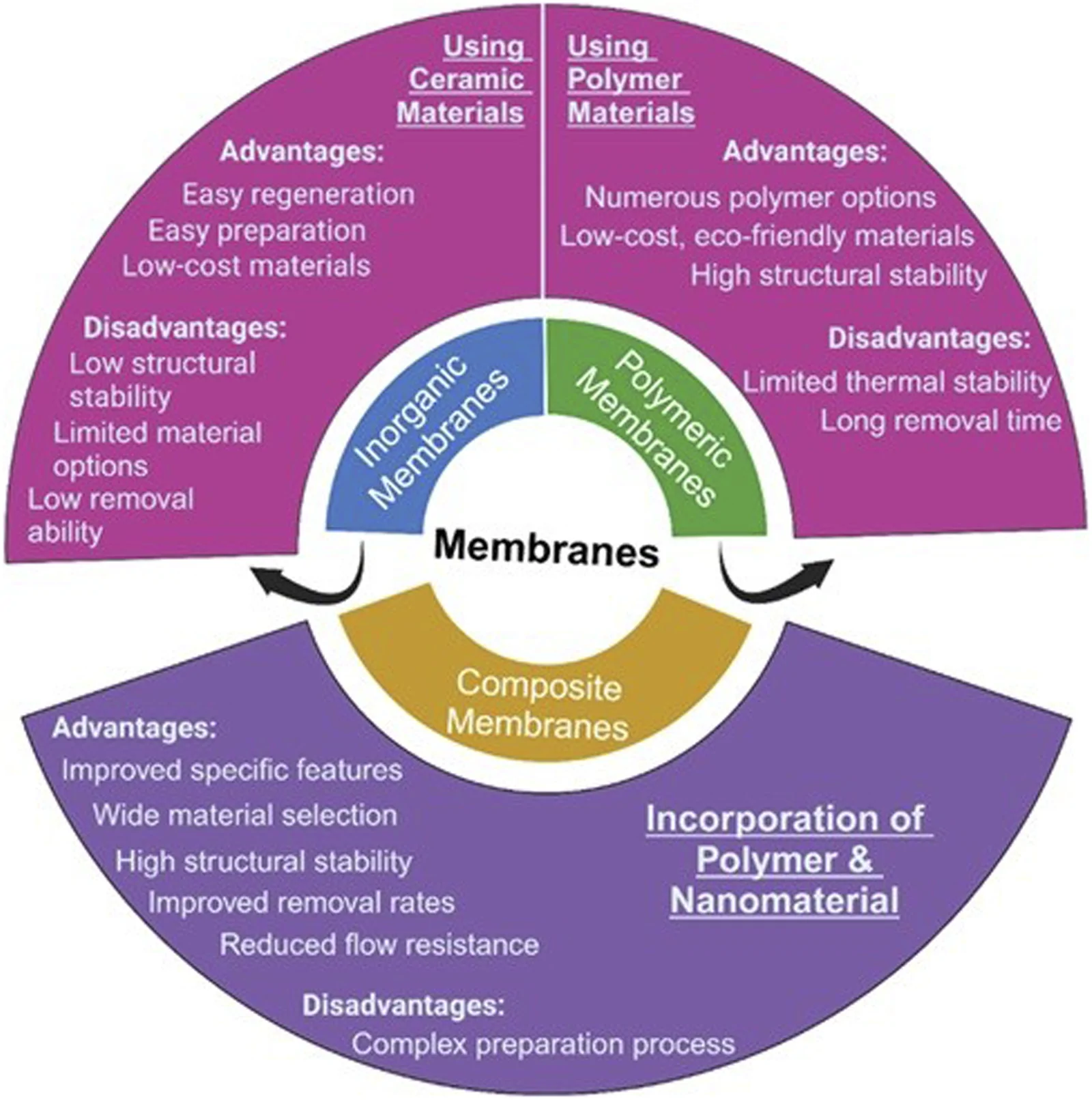
Typical membranes utilized for water/wastewater purification.

Polymeric membranes, such as those based on PA and PAN, offer structural tunability and ease of functionalization, which facilitate NF incorporation. Nevertheless, their viscoelastic nature may lead to surface rearrangement, pore collapse, or gradual degradation of NF–polymer interfaces under hydraulic pressure, temperature fluctuations, or long-term chemical exposure. These dynamic structural changes can progressively shield or deactivate NF active sites, diminishing hydrophilicity, surface charge density, and catalytic functionality over time.

Composite membranes, including inorganic–organic hybrid systems, represent another promising platform for NF integration, combining the structural robustness of inorganic materials and the functional tunability of polymers. These hybrid systems enable precise control over surface chemistry, pore architecture, and interfacial properties, resulting in highly efficient filtration systems.^39^ Synergistic interactions between NFs and hybrid substrates have been further explored in applications such as oil–water separation and biocatalysis.^11^ Moreover, innovative strategies, including NF-modified carbon nanotubes, have expanded the applicability of hybrid membranes, enhancing their catalytic performance and selectivity for complex water treatment challenges.^73^

Despite these advances, several critical scientific and engineering gaps remain in the optimization of membrane materials for NF integration. A major challenge lies in the intrinsic mismatch between the hierarchical, metastable morphology of NFs and the physicochemical behavior of the membrane substrates that support them. Composite substrates introduce additional complexity. Achieving synergistic performance requires precise control over interfacial morphology, pore connectivity, and chemical gradients across hybrid layers. Most existing studies rely on laboratory-scale fabrication methods that fail to capture defect formation, phase segregation, and thermal mismatch stresses that are likely to occur during large-scale production. Furthermore, interaction between NF functionality and hybrid matrix dynamics under real wastewater compositions, including competing ions, natural organic matter, multivalent cations, surfactants, and biofilm precursors, remains insufficiently understood. Material selection strategies rarely integrate life cycle, environmental, or regulatory considerations. For example, the long-term leaching behavior of metal-phosphate or metal-oxide NFs, their transformation into secondary nano- or ionic species, and their interactions with aquatic biota are not yet comprehensively evaluated. Similarly, the environmental footprint associated with ceramic sintering, high-purity precursors, and solvent-intensive hybrid fabrication processes is often overlooked.

Overall, while polymeric, inorganic, and composite substrates each provide distinct advantages, the scientific basis for selecting optimal membrane material for specific NF architectures remains incomplete. Future progress will require multiscale investigations that integrate interfacial chemistry, mechanical stability, fouling dynamics, and environmental safety under realistic operating conditions and scalable manufacturing processes. Only through this deeper mechanistic understanding can NF-based membranes transition from promising research materials to robust, commercially deployable water purification technologies. No single membrane material can currently satisfy all practical requirements for water purification. Polymeric membranes generally offer superior processability and lower fabrication costs, whereas ceramic membranes provide greater chemical and mechanical stability. Hybrid membrane systems appear to offer the greatest potential by combining the advantages of both materials; however, further optimization of NF–polymer interfacial compatibility and large-scale manufacturing remains necessary before widespread commercialization can be achieved.

### 2.4. Local membrane properties: how NF-based membranes work

NF-based membranes operate by integrating functional nanomaterials that modify the local physicochemical properties of filtration surfaces. Through tailored control of hydrophilicity, mechanical strength, and reactive surface interactions, these membranes achieve enhanced filtration performance. Several studies have shown that NFs incorporation increases water flux, reduces biofouling, and improves contaminant rejection rates across diverse membrane platforms.^74^

A defining advantage of NF-based membranes is their ability to facilitate selective adsorption and catalytic degradation of pollutants. Metal oxide NFs, such as ZnO and CeO_2_, introduce antimicrobial and photocatalytic functionalities that significantly improve water treatment efficiency.^26^ Their high surface reactivity and tunable pore structures further facilitate the removal of pharmaceuticals, heavy metals, and other emerging contaminants.^75^

NFs also contribute to enhanced antifouling properties by creating self-cleaning surfaces that inhibit organic and microbial buildup. This functionality is particularly valuable in long-term filtration applications, where membrane longevity and operational stability are critical.^7^ By leveraging these properties, NF-based membranes have demonstrated potential in advanced filtration systems, particularly for desalination and wastewater treatment.^20^

Beyond surface effects, NF-membrane interactions can improve mechanical robustness and stability under high-pressure operation. This has been particularly significant in industrial wastewater treatment and pharmaceutical purification, where membranes must withstand sustained hydraulic stress.^39^

Recent studies further reveal that NF incorporation can fundamentally alter the electronic and charge-transfer environment at the membrane surface, resulting in accelerated reaction kinetics and improved pollutant capture. Heterostructure NFs, such as carbon quantum dot-decorated ZnO NFs grown directly on nanofibrous membranes, create highly active photocatalytic interfaces that enhance light absorption, promote charge separation, and enable rapid degradation of organic contaminants under visible-light irradiation. For example, photocatalytic degradation reported at approximately 88–92% for the CQD–ZnO NF membrane. The hierarchical petals increase catalytic site density while facilitating directional electron transport, thereby strengthening oxidative reactions and enabling self-regeneration of the membrane surface during operation.^26^

Similarly, NF-substrate heterojunctions such as CoNiSe_2_ NFs anchored on layered supports exhibit built-in electric fields (BIEF) arising from interfacial work function differences. These internal fields promote electron redistribution and optimize the adsorption–desorption energetics and enhance selective pollutant capture and catalytic transformation. The radially aligned nanosheets form interconnected mesoporous channels that improve mass transfer, lower flow resistance, and support higher permeability without compromising rejection performance.^76^

Other NF systems, such as Mo-modified NiFe LDH NFs, demonstrate how top-down etching and interlayer modification generate high-valence metal sites, oxygen-rich surface, and abundant defect-rich regions. When integrated into membranes, these features strengthen adsorptive interactions with charged and polar contaminants, improve wettability, and provide stronger chemical anchoring to the membrane matrix. Such NFs also exhibit superior mechanical resilience and structural integrity under cyclic hydraulic stresses encountered in industrial processes.^31^

Moreover, NF architectures grown directly on electrospun or porous substrates, such as LDH-wrapped fibers, ZnO NF/SiC composites, and hybrid Cu_3_(PO_4_)_2_-based NFs, exhibit exceptional structural stability and resistance to particle leaching. Their anchored hierarchical growth distributes mechanical loads, preserves pore integrity, and introduces organized nanochannels that support rapid water transport and selective sieving. These properties further elevate the contaminant removal efficiency of the composite membrane.

Collectively, these findings demonstrate that NFs do more than simply increase surface roughness or introduce reactive sites. Instead, they engineer the local membrane microenvironment, enabling synergistic improvements in wettability, charge transport, catalytic activity, pore architecture, and mechanical endurance. This multifunctional impact underscores the growing recognition of NF-enabled membranes as next-generation filtration platforms capable of addressing emerging contaminants while maintaining high-flux and long-term stability under demanding wastewater treatment conditions.

## 3. Recent developments in NF-based membranes for water and wastewater treatments

### 3.1. Recent studies

NFs have recently emerged as a highly promising nanostructured material for enhancing membrane performance in water treatment. These hierarchical, flower-like nanostructures exhibit high surface area, superior adsorption capacities, and improved catalytic functionalities. Their integration into membranes has primarily targeted improvements in hydrophilicity, antifouling performance, and mechanical stability. NFs synthesized from metal oxides, carbon-based materials, and hybrid composites have been successfully incorporated into both polymeric and ceramic membranes, demonstrating improved permeability and selective pollutant removal efficiency. For instance, titanium dioxide (TiO_2_) NFs integrated into PVDF membranes enhanced photocatalytic degradation of organic contaminants while maintaining high flux stability. Similarly, zinc oxide (ZnO) NFs embedded in PSf membranes improved hydrophilicity and reduced biofouling tendencies, extending operational lifespan. These studies collectively highlight the potential of NFs to advance next-generation membrane systems for sustainable water treatment.^77^

Composite materials consist of two or more active components with distinct physical and chemical properties have further expanded NF applications. Nano-enhanced composite membranes (NECMs) integrate nanoscale fillers with polymeric barrier layers, providing tailored performance for water and wastewater treatment. Typically, these membranes feature a polymer support layer at the base and a functional NF-enriched upper layer.^24,56,78^

### 3.2. Application-based case studies at lab scale

Laboratory-scale studies have demonstrated the versatility and efficacy of NF-enhanced membranes in treating various types of wastewater streams. Copper oxide (CuO) NF-coated membranes showed significant improvement in heavy metal ion removal due to their high adsorption affinity and electrostatic interactions.^79^ Cobalt-based NFs incorporated into membrane bioreactors (MBRs) enhanced microbial attachment, aiding biofouling control and stabilizing permeate quality.^77,80^ Graphene oxide (GO) NFs integrated into ultrafiltration membranes reduced irreversible fouling by 50% and enhanced water permeability compared to conventional polymeric membranes.^77^ Silver (Ag) NF-embedded membranes exhibited strong antibacterial properties, reducing biofilm formation and extending membrane operational life.^81^ These laboratory-scale experiments underscore the versatility and potential of NFs in membrane applications. The findings support further development toward pilot-scale evaluations for real-world implementations.

Additional studies underscore the broad functional versatility of NFs across diverse materials. Metal–organic framework (MOF)-derived NFs were incorporated into PA and PVDF substrates to improve hydrophilicity and surface charge density, improving water permeation and reducing organic fouling in the filtration of dye-rich effluents. Covalent organic framework (COF) NFs created super-hydrophilic separation layers for efficiently treating oil–water emulsions, with hierarchical petals promoting rapid water wetting and minimizing oil adhesion. Carbon-based NFs enhanced adsorption-driven removal of pharmaceutical residues, benefiting from their large external surface area and rapid contaminant diffusion pathways. Metal phosphate and metal oxide NFs further improved removal of charged pollutants, such as heavy metals and oxyanions, through combined electrostatic and surface complexation mechanisms. Collectively, these studies demonstrate that NFs, whether carbonaceous, inorganic, or hybrid, provide tunable surface chemistry, hierarchical porosity, and catalytic or adsorptive functionality that significantly elevate membrane performance across a wide spectrum of wastewater types.^20,31,48,76,82^

A recent investigation on Black@ZnO–Gr-modified carbon fiber membranes highlighted the benefits of hierarchical ZnO NF structures in degreasing wastewater treatment. The ZnO–graphene coating enhanced hydrophilicity, adsorption capacity, and photocatalytic self-cleaning, enabling efficient oil and organic pollutant removal while maintaining strong antifouling stability.^44^Fig. 7 through 8 and Table 3 summarize representative NF synthesis pathways and membrane architectures developed in recent studies, illustrating the diversity of fabrication methods and structural configurations.

**Fig. 7:**
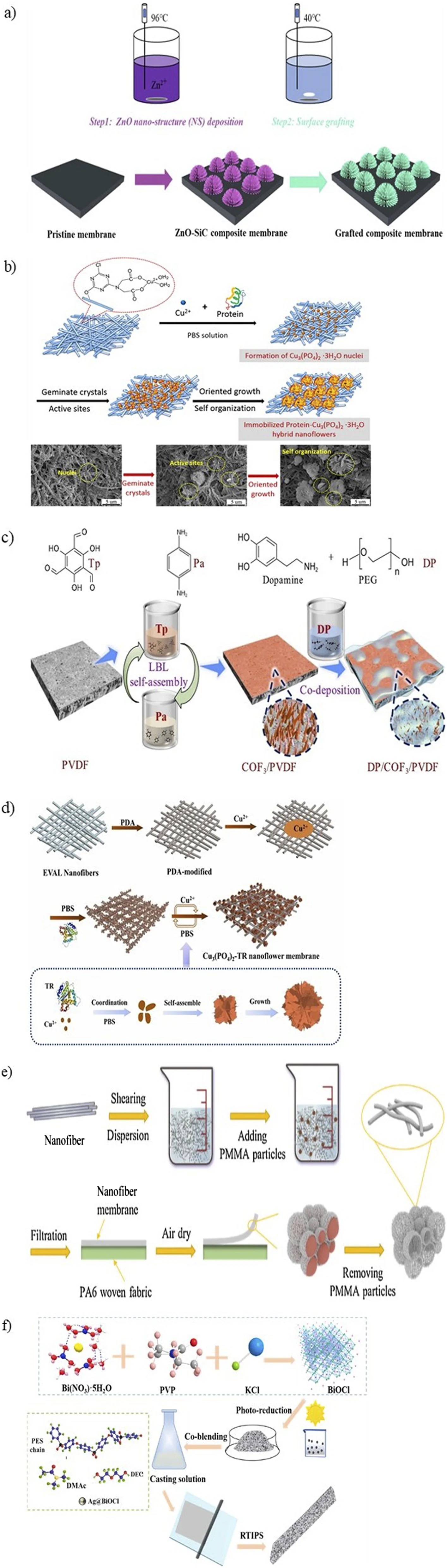
Fabrication of some NF-based membrane (a) schematic illustration of the ZnO NF@SiC membrane preparation.^7^ (b) Growing mechanism of Cu_3_(PO_4_)_2_·3H_2_O–protein nanoflowers^84^ (c) fabrication process of the DP/COF3/PVDF membrane, utilizing LBL self-assembly and co-deposition techniques.^34^ (d) Fabrication of the NF-embedded EVAL nanofiber layer and the growth of Cu_3_(PO_4_)_2_–TR hybrid NF.^11^ (e) Fabrication process of the hierarchically porous nanofibrous membrane.^39^ (f) Preparation of ultrahigh antifouling ultrafiltration membrane based on Ag NPs photoreduction modified PVP/BiOCl NF for efficient membrane fouling removal.^62^ Adapted with permission from ref. 7, 11, 34, 39, 62 and 84. Copyright permissions obtained from the respective copyright holders.

**Fig. 8:**
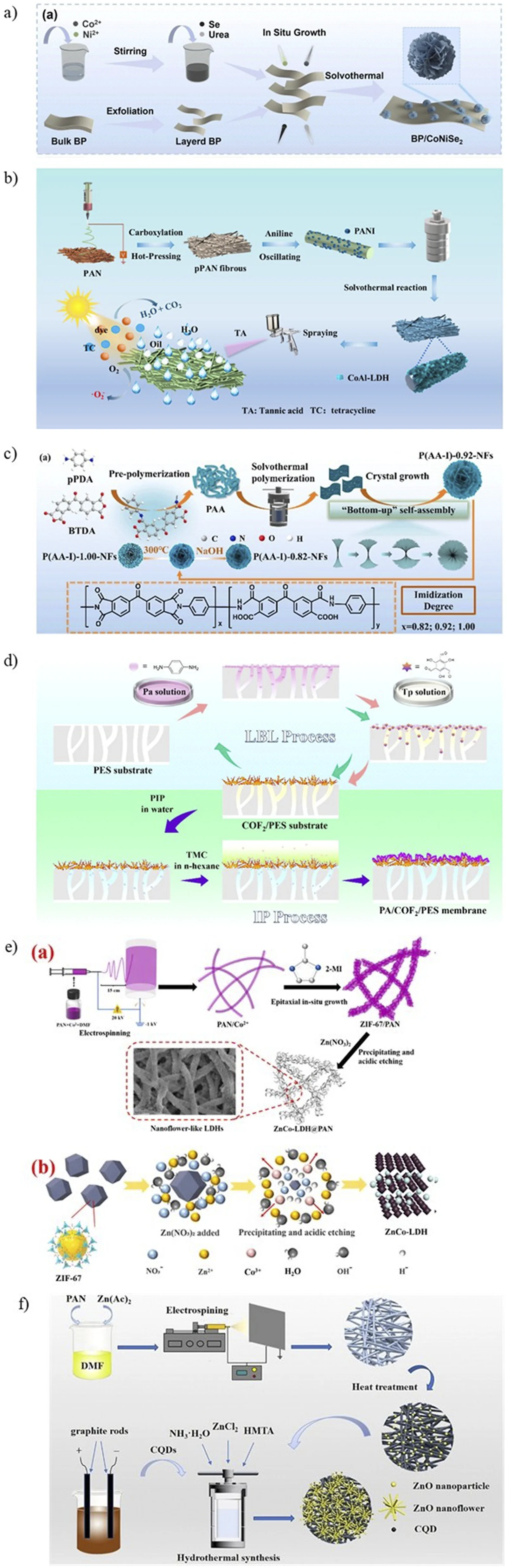
Fabrication of some NF-based membrane (a) fabrication of some NF-based membrane. Schematic illustration for the synthesis of BP/CoNiSe_2_.^76^ (b) Schematic diagram of the heterogeneous CoAl-LDH@PANI/pPAN fibrous composite membrane.^83^ (c) Schematic diagram of the preparation process of self-assembled NF-mediated interfacial polymerization through tunable intra- and inter-layer channels.^74^ (d) Schematic illustration of the preparation process of PA/COF2/PES membranes.^20^ (e) Illustration of the preparation procedure for the ZnCo-LDH@PAN membrane and formation schematic of ZnCo-LDH nanoparticles on PAN nanofibers.^75^ (f) Schematic diagram of carbon quantum dots-decorated ZnO heterostructure NFs grown on nanofiber membranes.^26^ Adapted with permission from ref. 20, 26, 74–76 and 83. Copyright permissions obtained from the respective copyright holders.

**Table 3:** Comparative summary of representative nanoflower-based membrane systems for water and wastewater treatment

Ref.	Nanoflower/loading	Membrane type	Feed composition	Filtration mode	Rejection/separation
7	ZnO nanoflower; loading NR	SiC ceramic	Water-in-oil emulsion	Pressure-driven filtration	∼98.9–99.2% water rejection
75	ZnCo-LDH nanoflower; loading NR	PAN nanofibrous membrane	Oil-in-water emulsion	Cross-flow filtration	>99%; up to 99.4%
39	Inorganic–organic hybrid nanoflowers; enzyme-loaded	3D porous nanofibrous membrane	Organic dye solutions	Membrane-supported catalytic treatment	High dye degradation
26	CQD-decorated ZnO heterostructure nanoflowers; loading NR	PAN nanofiber membrane	Methylene blue solution	Photocatalytic membrane treatment	92.08% degradation under UV; 88.36% under sunlight
11	Cu_3_(PO_4_)_2_–trypsin nanoflower; 282.1 ± 18.2 µg cm^−2^	EVAL nanofiber membrane	β-Lactoglobulin solution	Permeation/tangential flow	98.1% degradation under permeation; 71.7% under tangential flow
34	COF nanoflowers; loading NR	Nanoflower-functionalized composite membrane	Oil-in-water emulsion	Vacuum filtration	>98%; 98.4% after 10 cycles
20	COF nanoflowers; loading NR	Nano-wrinkled polyamide membrane	Salt solution + antibiotics	Pressure-driven membrane separation	Desalination and antibiotic rejection
83	CoAl-LDH@PANI nanoflower; loading NR	PAN nanofibrous membrane	Organic contaminants and emulsified wastewater	Photocatalytic membrane filtration	Organic contaminant degradation + oil/water separation
62	Ag@BiOCl nanoflower; loading NR	PES ultrafiltration membrane	Humic acid solution	Ultrafiltration/visible-light self-cleaning	∼95% HA rejection
82	TiO_2_ nanoflower–calcium bentonite; loading NR	Hollow-fiber microfiltration membrane	Real refinery-produced water	Photocatalytic–membrane treatment	Refinery-water contaminant removal
84	Copper-based protein nanoflowers; loading NR	Nanoscale fibrous membrane	Enzymatic/catalytic model feed	Membrane-supported catalytic treatment	Catalytic treatment performance

Collectively, these studies demonstrate that NF-based membranes consistently improve membrane performance compared with unmodified membranes; however, the reported improvements vary considerably because of differences in nanoflower composition, membrane fabrication methods, operating conditions, and feed-water characteristics. Consequently, direct comparison among published studies remains challenging. Recent research indicates a clear shift from single component nanoflowers toward multifunctional hybrid architectures that simultaneously integrate adsorption, photocatalysis, and antifouling properties. Nevertheless, standardized testing protocols and long-term performance evaluations are still lacking, limiting the ability to identify the most effective membrane designs for practical applications.

## 4. Scalability of fabrication techniques

### 4.1. Scope: large-scale application

NF-based membranes exhibit exceptional potential in laboratory-scale water and wastewater treatment due to their unique structural properties, including large surface area, higher adsorption capabilities, and improved catalytic activity. However, translating these laboratory innovations into commercially viable, large-scale applications present multiple technical and economic challenges.

A primary hurdle is the development of scalable and cost-effective fabrication methods. Current techniques, including electrochemical deposition and template-assisted growth, face limitations in producing uniform NF structures across large membrane areas.^77^ Roll-to-roll manufacturing and continuous production processes show promise in achieving consistent membrane quality and large-scale output.^17^ Electrospinning is emerging as a viable large-scale production method, producing fibrous nanostructures with tunable porosity to enhance water filtration efficiency.^67^

Beyond the scalable synthesis of nanoflowers, the development of robust membrane architectures is equally important for successful industrial implementation. One promising strategy is the incorporation of nanoflower-based materials into multilayer non-woven sandwich structures, where the active nanocomposite is immobilized between mechanically robust porous substrates. Such architectures provide enhanced structural integrity, minimize nanomaterial detachment and leaching, improve handling during continuous-flow operation, and facilitate replacement or regeneration of the active layer. Furthermore, non-woven supports are compatible with established textile and roll-to-roll manufacturing technologies, making them attractive for large-scale membrane fabrication. A recent study by Hassan *et al.* demonstrated this concept using a non-woven Nomex sandwich filter containing an activated carbon/polypyrrole (AC/PPy) nanocomposite fabricated by hot pressing. The multilayer filter exhibited excellent mechanical stability, prolonged operational durability, and reusability while maintaining high contaminant removal performance, illustrating how sandwich architectures can facilitate the practical immobilization of functional nanocomposites in scalable filtration systems.^85^

Although this approach has primarily been demonstrated using activated carbon/polymer composites, the same design principle could be extended to nanoflower-based membranes, where non-woven sandwich configurations may improve nanoflower immobilization, reduce catalyst leaching, enhance mechanical durability, and support pilot- and industrial-scale water treatment applications.

### 4.2. Challenges for real-world deployment

Despite promising advances in laboratory-scale studies, several barriers must be overcome before NF-based membranes can be applied in full-scale water treatment facilities. One significant concern is scalability and fabrication costs. Producing NFs with uniform morphology and controlled functionalization at industrial scale remains challenging.^77,86^ Optimization of fabrication cost and mass production efficiency is essential for commercial viability. Another challenge is the stability and durability of NF-based membranes. NF-enhanced membranes must maintain structural integrity under high pressures, fluctuating pH levels, and dynamic ionic environments. Long-term studies are needed to evaluate performance retention under operational conditions.^62,87^ Membrane fouling and compatibility issues also remain a barrier to real-field applications. While NFs improve antifouling performance, irreversible fouling mechanisms, such as organic and biofouling in real wastewater environments, require further investigation.^73,77,86^ Furthermore, environmental and health concerns need to be thoroughly addressed. Potential NF leaching into treated water poses environmental and human health risks.^88^ Understanding the fate, transformation, and toxicity of released NFs is crucial for regulatory approvals and safe deployment. Addressing these challenges through continued research and development will be critical to unlocking the full potential of NF-enhanced membranes in real-world water treatment applications.

In addition to treatment performance, the environmental safety of nanoflower-based materials should be considered during repeated use and regeneration. Changes in pH, ionic strength, natural organic matter, and cleaning conditions may influence nanoflower stability and potentially promote metal dissolution or particulate detachment. Therefore, long-term studies should evaluate material stability over multiple treatment and cleaning cycles, together with ecotoxicological assessment of potentially released species. Appropriate recovery and end-of-life management strategies should also be considered to minimize uncontrolled release of nanomaterials into aquatic environments and to support their safe implementation at larger scales.^89^

Although recent publications increasingly report excellent laboratory-scale performance, relatively few studies include pilot-scale demonstrations, continuous-flow experiments, or comprehensive techno-economic analyses. Future research should therefore focus on validating membrane performance under realistic operating conditions while simultaneously evaluating manufacturing cost, environmental sustainability, and long-term operational reliability.

#### 4.2.1 Mechanical and chemical stability

Maintaining mechanical integrity and chemical stability is crucial for industrial applications. NF membranes may exhibit brittleness or structural degradation under high pressures and extreme pH.^24^ Strategies such as hybridizing NFs with robust support materials (*e.g.*, MOFs and polymer composites) and applying surface cross-linking techniques have shown promise in enhancing durability.^88^ For example, embedding Cu_3_(PO_4_)_2_ NFs within a nanofiber support layer improves mechanical strength while maintaining high permeability.^39^

#### 4.2.2 Fouling mitigation

Membrane fouling remains a significant bottleneck in scaling NF membranes. Organic, inorganic, and biological matter accumulation reduces permeability and increases maintenance costs. Mitigation strategies include incorporating antifouling agents such as Ag nanoparticles and titanium dioxide (TiO_2_), as well as enhancing surface hydrophilicity.^57^ Hybrid NF membranes incorporating photocatalytic nanomaterials have demonstrated self-cleaning capabilities under light exposure, further suppressing fouling.^62^

Although contact angle measurements provide useful information regarding membrane hydrophilicity, they alone are insufficient to demonstrate durable antifouling performance. A comprehensive evaluation should include quantitative metrics such as normalized flux decline (NFD), reversible and irreversible fouling ratios (*R*_r_ and *R*_ir_), flux recovery ratio (FRR), filtration duration, cleaning efficiency, membrane stability, and performance over repeated fouling–cleaning cycles. A limited number of studies have comprehensively reported these parameters. For example, Ag@BiOCl nanoflower-modified ultrafiltration membranes achieved an FRR of 85.98% following visible-light-assisted self-cleaning while simultaneously reporting reversible and irreversible fouling ratios, silver leaching, and antibacterial performance, providing a comprehensive assessment of membrane durability.^37,39,62^ Similarly, nanoflower-branched ZnCo-LDH/PAN membranes exhibited an FRR of 97.8% and maintained stable separation performance over 10 consecutive filtration cycles, demonstrating excellent chemical and mechanical stability. Other promising systems, including nano-wrinkled polyamide membranes incorporating covalent organic framework (COF) nanoflowers, ZnO nanoflower@SiC ceramic membranes, and enzyme-immobilized nanofibrous membranes, also demonstrated enhanced permeability, fouling resistance, and operational stability during prolonged filtration or repeated reuse.^7,20,58,83^ However, many studies primarily reported reductions in contact angle or improvements in surface hydrophilicity without quantitatively evaluating long-term fouling behavior, making direct comparison of membrane durability challenging. Collectively, these findings indicate that improved hydrophilicity alone should not be interpreted as evidence of durable antifouling behavior. Future investigations should therefore adopt standardized antifouling evaluation protocols that integrate surface characterization with quantitative performance metrics, including FRR, normalized flux decline, reversible and irreversible fouling ratios, cleaning efficiency, long-term filtration stability, and mechanical durability, to enable meaningful comparison and accelerate the practical development of nanoflower-based membrane systems.

#### 4.2.3 Economic viability and market integration

The economic feasibility of NF-enhanced membranes is influenced by fabrication costs, operational efficiency, and maintenance requirements. Although these membranes demonstrate superior performance, high production costs and specialized fabrication infrastructure requirements hinder widespread adoption.^24,59^ Collaborative research and industry partnerships are essential to develop cost-effective fabrication methods and pilot-scale demonstrations. Enhancing membrane longevity and reducing maintenance through robust hybrid designs are key to achieving economic viability and market competitiveness.^67,90^

## 5. Future directions and opportunities

Future research should focus on optimizing NF structural properties to enhance selectivity and permeability while maintaining mechanical stability. Innovations in membrane surface engineering, including bio-inspired coatings and hybrid material integration, offer promising strategies to mitigate fouling and improve structural robustness.^91^ Furthermore, the adoption of advanced synthesis techniques, such as 3D printing and scalable nanofiber fabrication, will play a significant role in overcoming current manufacturing limitations.^63^ The integration of computational modeling and AI-driven design can further accelerate material discovery by predicting processing–structure–property relationships of NFs and composites with their incorporation before experimental synthesis, reducing development time and cost.

Artificial intelligence (AI) and machine learning (ML) are expected to become indispensable tools for the next generation of nanoflower-based membrane development. Recent advances demonstrate that data-driven approaches can establish quantitative relationships between membrane composition, nanostructure, fabrication parameters, and separation performance, enabling the prediction of permeability, selectivity, fouling resistance, and long-term stability while substantially reducing experimental effort. Modern ML workflows integrate experimental data, molecular descriptors, computational simulations, and high-throughput screening to accelerate membrane material discovery and optimization. Explainable AI techniques, such as SHAP analysis and partial dependence plots, further reveal the influence of key structural parameters—including pore size, surface chemistry, functional groups, nanoparticle loading, and zeta potential—on membrane performance, thereby providing valuable design guidelines rather than merely predictive models. More recently, generative AI, large language models, inverse design strategies, and physics-informed machine learning have emerged as promising approaches for discovering novel membrane materials with targeted properties by coupling computational prediction with experimental validation. For nanoflower-functionalized membranes, these emerging methodologies could significantly accelerate the optimization of nanoflower morphology, composition, interfacial interactions, and membrane fabrication conditions, leading to multifunctional membranes with improved permeability, selectivity, antifouling properties, and operational stability. Despite these advances, challenges associated with limited standardized datasets, model interpretability, and the integration of multi-scale experimental and computational data remain barriers to widespread implementation. Future research should therefore emphasize the development of open membrane databases, hybrid physics-informed AI frameworks, and autonomous high-throughput experimentation to enable the rational design of next-generation nanoflower-based membranes for sustainable water treatment and environmental remediation.^92–94^

Future studies should further evaluate the environmental safety and long-term stability of the BC–NF material. In particular, potential copper release should be quantified using ICP-OES or ICP-MS under relevant water-treatment conditions, while complementary particle characterization should be performed to distinguish dissolved metal release from particulate detachment. Metal and particle mass-balance analyses would provide further information on the stability of the nanoflowers and the fate of copper during treatment. Repeated cleaning and regeneration experiments should also be conducted to determine whether material reuse promotes copper leaching or structural deterioration. In addition, ecotoxicological assessment and appropriate recovery and end-of-life disposal strategies should be investigated before considering larger-scale application of the material.^95,96^

In conclusion, NF-based membranes offer promising possibilities for water and wastewater treatment; however, addressing challenges related to scalability, stability, fouling, and economic viability is equally important. Continuous-flow hydrothermal or solvothermal synthesis, additive manufacturing, and electrospinning approaches can enable uniform, large-scale production of NF membranes with precise architecture. Evaluating chemical stability under extreme pH, oxidizing environments, and high-pressure operation, along with life-cycle analysis and cost modeling, will ensure the membranes are both durable and economically viable. Ultimately, interdisciplinary collaboration among material scientists, chemical engineers, environmental researchers, and data scientists will be pivotal in translating NF-based membranes from laboratory innovation to industrial-scale application, unleashing their full potential in sustainable and energy-efficient water treatment systems. Current research trends indicate a gradual transition from empirical membrane development toward data-driven materials engineering. Machine learning, high-throughput computational screening, and digital process optimization are expected to accelerate the identification of optimal NF morphologies, compositions, and fabrication conditions. These approaches may substantially reduce experimental costs while enabling the rational design of multifunctional membranes with improved permeability, selectivity, durability, and environmental safety.

## 6. Conclusion

Three-dimensional nanoflower (NF)-based membranes have emerged as a new generation of multifunctional membrane materials capable of addressing several long-standing challenges in water and wastewater treatment. Their hierarchical flower-like architectures provide high surface area, abundant exposed active sites, interconnected transport pathways, and tunable surface chemistry, enabling simultaneous enhancement of adsorption, membrane separation, photocatalytic activity, hydrophilicity, antifouling behavior, and contaminant selectivity. Unlike conventional nanomaterials, the unique three-dimensional morphology of NFs promotes improved interfacial compatibility with membrane matrices, efficient mass transport, and higher accessibility of reactive sites, and overall membrane performance.

This review critically demonstrates that the performance of NF-based membranes is governed not only by their chemical composition but also by the intricate relationships among nanoflower morphology, petal thickness, defect density, surface functionality, interfacial adhesion, and membrane fabrication strategy. The literature consistently shows that rational engineering of these structural characteristics can simultaneously improve water permeability, contaminant rejection, catalytic efficiency, and fouling resistance while mitigating the traditional permeability–selectivity trade-off. Nevertheless, the reported performance improvements vary considerably because of differences in synthesis methods, membrane substrates, operating conditions, and characterization protocols, highlighting the urgent need for standardized evaluation procedures that enable meaningful comparison across different studies.

Despite remarkable laboratory-scale progress, several fundamental challenges continue to limit the practical implementation of NF-based membranes. Long-term structural stability under realistic operating conditions, nanoflower detachment and metal leaching, membrane durability during repeated cleaning cycles, and performance in complex water matrices containing natural organic matter, competing ions, microorganisms, and suspended solids remain insufficiently understood. Furthermore, most published studies rely on short-term experiments using simplified synthetic feed solutions, while relatively few investigations have evaluated pilot-scale operation, long-term membrane performance, environmental safety, life-cycle sustainability, or techno-economic feasibility. Addressing these knowledge gaps will be essential for translating laboratory achievements into reliable industrial technologies.

Future progress will depend on integrating advanced materials engineering with computational and data-driven approaches. Artificial intelligence, machine learning, molecular simulations, and digital modeling provide unprecedented opportunities to accelerate nanoflower design, predict membrane performance, optimize fabrication parameters, and establish quantitative structure–property–performance relationships. When combined with scalable manufacturing techniques such as continuous hydrothermal synthesis, electrospinning, additive manufacturing, and roll-to-roll membrane production, these emerging technologies have the potential to significantly reduce development time while improving reproducibility, cost-effectiveness, and commercial scalability.

Overall, 3D NF-based membranes represent a highly promising and rapidly evolving platform for sustainable water purification. Their ability to integrate multiple treatment mechanisms within a single membrane architecture offers significant opportunities for developing energy-efficient, high-performance, and environmentally sustainable separation technologies. Future research should move beyond maximizing laboratory-scale performance toward establishing standardized testing protocols, validating long-term operation under realistic conditions, ensuring environmental safety, and developing economically viable manufacturing strategies. Achieving these objectives will require close collaboration among materials scientists, membrane engineers, environmental researchers, computational scientists, and industrial stakeholders. Such interdisciplinary efforts will ultimately enable the successful transition of NF-based membranes from promising laboratory materials to robust, commercially deployable technologies capable of addressing global water and wastewater treatment challenges.

## Conflicts of interest

There are no conflicts of interest to declare.

## Data Availability

No primary research results, software or code have been included and no new data were generated or analysed as part of this review.
